# Computational guided approach for drug repurposing against SARS-CoV-2

**DOI:** 10.2217/fvl-2020-0403

**Published:** 2021-03-02

**Authors:** Jigisha Anand, Tanmay Ghildiyal, Aakanksha Madhwal, Rishabh Bhatt, Devvret Verma, Nishant Rai

**Affiliations:** 1 ^1^Department of Biotechnology, Graphic Era (Deemed to be University), Clement Town, Dehradun, Uttarakhand-248001, India; 2 ^2^Department of Life Sciences, Graphic Era (Deemed to be University), Clement Town, Dehradun, Uttarakhand-248001, India

**Keywords:** ADMET, *in silico*, nsp3, nsp5, protein modeling, SARS-CoV-2

## Abstract

**Background:** In the current SARS-CoV-2 outbreak, drug repositioning emerges as a promising approach to develop efficient therapeutics in comparison to *de novo* drug development. The present investigation screened 130 US FDA-approved drugs including hypertension, cardiovascular diseases, respiratory tract infections (RTI), antibiotics and antiviral drugs for their inhibitory potential against SARS-CoV-2. **Materials & methods:** The molecular drug targets against SARS-CoV-2 proteins were determined by the iGEMDOCK computational docking tool. The protein homology models were generated through SWISS Model workspace. The pharmacokinetics of all the ligands was determined by ADMET analysis. **Results:** The study identified 15 potent drugs exhibiting significant inhibitory potential against SARS-CoV-2. **Conclusion:** Our investigation has identified possible repurposed drug candidates to improve the current modus operandi of the treatment given to COVID-19 patients.

Coronaviruses (family: *Coronaviridae*) are known to cause a variety of diseases in mammals and birds. Due to their capability of infecting a large array of animal hosts, these viruses can be transmitted between species. SARS outbreak in southern China (2002) and Middle East respiratory syndrome (MERS) outbreak in the Middle East (2012) were both caused by (human) coronaviruses. SARS-CoV-2, another zoonotic (bat-borne), positive sense, ssRNA coronavirus, and a successor of SARS-CoV-1 (SARS outbreak), is responsible for the current COVID-19 crisis [1]. It belongs to the subfamily Orthocoronavirinae; genus *Betacoronavirus* (lineage B) which naturally infects bats and rodents [2–4].

The genome of SARS-CoV-2 consists of seven genes organized into 5′ nonstructural protein-coding regions and 3′ structural and nonessential accessory protein-coding regions [5]. Gene 1 is organized into two very large open reading frames (1a and 1b), which are translated into replicase consisting of two large polypeptides – pp1a (∼400 kDa) and pp1b (∼800 kDa). The replicase comprises of protein domain that contains 16 nonstructural protein units indicated by nsp1–nsp16 [6]. These nsp1–nsp16 form double membrane vesicles which is a virion replication and transcription complex [7], while nonstructural protein 3 (nsp3) plays an important role in the virion structure, replication and transcription [8].

Genes 2–7 are translated from subgenomic mRNA which encodes the major viral structural proteins like spike protein (S), envelope protein (E), membrane protein (M), nucleocapsid protein (N) and accessory proteins that are essential for the virus–cell receptor binding. The newly synthesized structural proteins are released into the endoplasmic reticulum. All of these proteins, along with N protein are linked to the viral genomic RNA and localized in the endoplasmic reticulum–golgi intermediate compartment (ERGIC) region from where they are packed in smooth-walled vesicles and released from the cell to infect other cells [9,10].

Consisting of 29,891 nucleotides encoding 9860 amino acids, the SARS-CoV-2 viral genome (26–32 kb) bears 82% similarity to the SARS CoV-1 genome [5]. Spike glycoprotein (S), membrane and envelope glycoproteins (M and E), and N protein are the major structural proteins encoded by this virus [6]. The spike glycoproteins that form the characteristic corona around the virus, form homodimers that bind to specific cellular receptors in the host via their N-terminus and are highly conserved regions [11]. The M glycoproteins play a pivotal role in helping the virus fusion into the cell, regeneration of virions and production of antigenic proteins [12,13].

The E glycoprotein of SARS-CoV-2 is a small protein composed of approximately 76–109 amino acids. About 30 amino acids in the N-terminal of E proteins allow attachment to the membrane of viruses and play a critical role in the assembly and morphogenesis of virions within the host cell [14,15]. N protein is a multifunctional protein that plays an important role in RNA packaging into nucleocapsid, replication and transcription [13]. This protein has shown 90% amino acid homology with SARS-CoV-2 responsible for causing an epidemic in 2003, and thus is highly conserved, stable and stands as a key target in antiviral drug development against SARS-CoV-2 [16].

The main protease (nsp5, 3CLpro) of SARS-CoV-2 shows the considerable sequence and structural resemblance with the protease of closely related members like SARS-CoV and MERS-CoV while it has dissimilarity with human protease [17]. This protein has a pivotal role in viral gene expression, formation of replication complex through its proteolytic cleavage of replicase polyproteins and induces cell death in uninfected neighboring cells [17–19]. Hence, viral protease is a well-studied SARS-CoV-2 protein and is a validated drug target for the development of novel coronavirus potential inhibitors. The main protease, therefore, could serve as the prime target to inhibit SARS-CoV-2 replication and preventing uncontrolled regulation of signaling cascade in infected cells, which may trigger death in neighboring uninfected cells [19].

The pathogenesis of SARS-CoV-2 initiates with its replication, which occurs in the host cell cytoplasm. The binding of the virus to the ACE-2 receptor on the host cell surface via receptor binding domain of spike (S) protein causes a conformational change in the precursor protein structure and thereby initiates the process of viral invasion in the host cell [20,21]. ACE-2 which is a membrane-bound zinc metalloenzyme and monocarboxypeptidase are found ubiquitously as surface receptor glycoproteins on heart, intestine, kidney and pulmonary alveolar (type II) cells of humans [22,23].

The infection and fatalities in coronavirus disease known as COVID-19 infection (also formerly called as 2019 novel coronavirus) are reportedly higher in individuals with comorbidities like hypertension, diabetes, liver and kidney dysfunctions [24]. This has imposed a challenging situation for health professionals to select appropriate therapeutics for the treatment under limited time for clinical setup and unavailability of new and specific drugs [25,26].

Therefore, because of the increasing numbers of patients with comorbidities and the associated risk factor linked with COVID-19 infections, drug repurposing has received a lot of interest from researchers. Drug repurposing is a process of identifying the novel application of approved drugs; this is feasible and cost-effective compared with the *de novo* drug-discovery mechanism [26,27].

Considering the prospects of repurposed drugs and structural relatedness of SARS-CoV-2 with SARS-CoV and MERS-CoV, we have selected 130 US FDA-approved drugs that have reportedly shown antiviral potentials. The selected drugs have been categorized into three types: antiviral drugs (designated as type-I); anticoagulants, antihypertensive drugs, and drugs against cardiovascular disease and respiratory tract infections (RTIs; designated as type-II); and other miscellaneous antimicrobial agents with prospects for drug repurposing (designated as type-III). Some of these drugs have been previously screened and repurposed for their application as an antiviral drug against SARS-CoV and MERS-CoV [26–28]. All 130 drugs were assessed for their possible inhibitory potential against target SARS-CoV-2 proteins using computational tools. The interaction energies generated via molecular docking study were determined. This study, therefore, provides possible drug repositioning candidates and their targets for future *in vitro* and *in vivo* investigations of SARS-CoV-2 therapeutics.

## Experimental study

**Figure 1.**
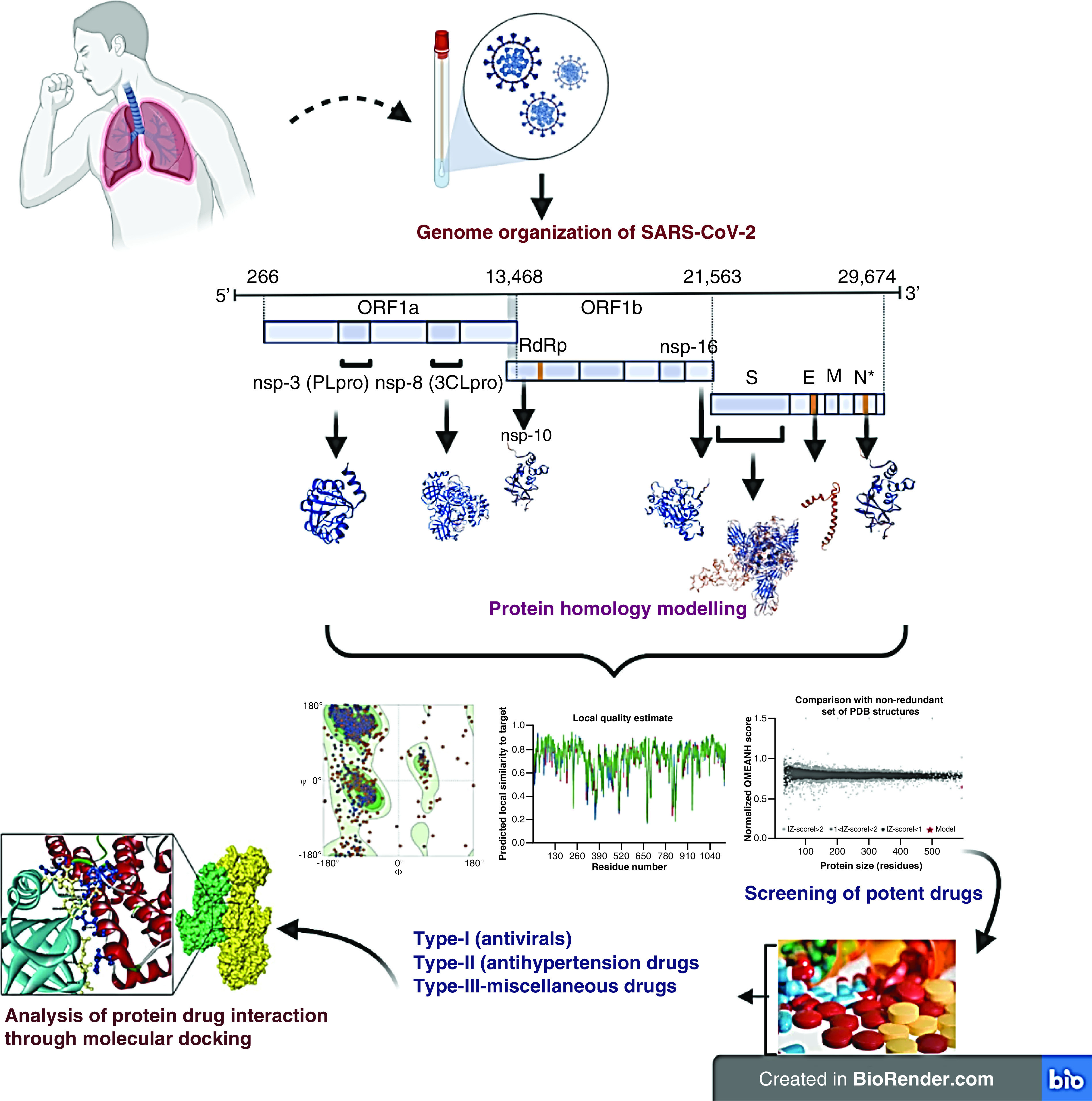
Graphical representation of work plan.

The course of our study was aimed at making use of *in silico* tools to screen the potential drugs with their inhibitory activity SARS-CoV-2. Based on the role of structural proteins including S, E, N, and nonstructural proteins nsp3(ribose phosphate), nsp5 (main protease), nsp10 and nsp16 (Mtase-methyltransferase) in the life cycle and pathogenesis of the SARS-CoV-2, we have performed their molecular docking with 130 FDA-approved drugs. The interaction of all the drugs and seven proteins were closely examined to identify the potential drug targets and possible novel candidates for drug repurposing in the treatment of COVID-19 infection (Figure 1).

## Materials & methods

### Retrieval of protein structures

The 3D structure of the structural and nonstructural proteins of SARS-CoV-2 were retrieved in protein data bank (PDB) format (.pdb) using the PHYRE program (Protein Homology/analogy Recognition Engine). The amino acid sequences were derived from the RCSB PDB in a FASTA format (www.rcsb.org/). The physicochemical and functional characterization of proteins was determined using Expasy’s Prot-param server [29]. The 3D-protein homology models were generated from the SWISS MODEL workspace [30,31]. The protein homology models were validated with a Ramachandran plot using the SWISS MODEL workspace. The stereochemical quality of the protein structures was assessed by PROCHECK to evaluate the presence of conserved sequences and related geometry of proteins [32].

#### Preparation of ligand molecules

The ligand molecules used in the study belong to different classes of medicinal drugs including: antiviral drugs (type-I); anticoagulants, antihypertensive drugs, and drugs against cardiovascular disease and RTIs (type-II); and other miscellaneous antimicrobial agents (type-III). The code of SMILES for all the aforementioned test molecules was procured from the online chemical database PubChem (https://pubchem.ncbi.nlm.nih.gov/) (Tables 1A, 1B & 1C). The 3D structures of the test molecules were then obtained by converting the SMILES code (.smiles format) into PDB (.pdb) format using chemical interconversion software Open Babel (v 2.3.1).

**Table 1A.** Type-I drugs used for *in silico* study against virulent proteins of SARS-CoV-2.

S. N.	Antiviral drugs	PubChem ID	Molecular formula	Molecular weight	2D structure
1.	Baloxavir marboxil	124081896	C_27_H_23_F_2_N_3_O_7_S	571.6 g/mol	
2.	Baricitinib	2 44205240	C_16_H_17_N_7_O_2_S	371.4 g/mol	
3.	Chloroquine	2719	C_18_H_26_ClN_3_	319.9 g/mol	
4.	Cobicistat	25151504	C_40_H_53_N_7_O_5_S_2_	776 g/mol	
5.	Danoprevir	11285588	C_35_H_46_FN_5_O_9_S	731.8 g/mol	
6.	Darunavir	213039	C_27_H_37_N_3_O_7_S	547.7 g/mol	
7.	Emtricitabine	60877	C_8_H_10_FN_3_O_3_S	247.25 g/mol	
8.	Favipiravir	492405	C_5_H_4_FN_3_O_2_	157.1 g/mol	
9.	Hydroxychloroquine sulfate	12947	C_18_H_28_ClN_3_O_5_S	434 g/mol	
10.	Lopinavir	92727	C_37_H_48_N_4_O_5_	628.8 g/mol	
11.	Remdesivir	121304016	C_27_H_35_N_6_O_8_P	602.6 g/mol	
12.	Ribavirin	37542	C_8_H_12_N_4_O_5_	244.2 g/mol	
13.	Ritonavir	392622	C_37_H_48_N_6_O_5_S_2_	720.9 g/mol	
14.	Sofosbuvir	45375808	C_22_H_29_FN_3_O_9_P	529.5 g/mol	
15.	Tenofovir	464205	C_9_H_14_N_5_O_4_P	287.21 g/mol	
16.	Umifenovir	131411	C_22_H_25_BrN_2_O_3_S	477.4 g/mol	

**Table 1B.** Type-II drugs used for *in silico* study against virulent proteins of SARS-CoV-2.

ACE inhibitors					
S. N.	Compound	PubChem ID	Molecular formula	Molecular weight	2D structure
1.	Enalapril	5388962	C_20_H_28_N_2_O_5_	376.4 g/mol	
2.	Captopril	44093	C_9_H_15_NO_3_S	217.29 g/mol	
3.	Lisinopril	5362119	C_21_H_35_N_3_O_7_	441.5 g/mol	
4.	Benazepril	5362124	C_50_H_60_Cl_2_N_4_O_13_S	1028 g/mol	
5.	Quinapril	54892	C_25_H_30_N_2_O_5_	438.5 g/mol	
6.	Perindopril	107807	C_19_H_32_N_2_O_5_	368.5 g/mol	
7.	Ramipril	5362129	C_23_H_32_N_2_O_5_	416.5 g/mol	
8.	Trandolapril	5484727	C_24_H_34_N_2_O_5_	430.5 g/mol	
9.	Fosinopril	23667962	C_30_H_46_NO_7_P	563.7 g/mol	
10.	Moexipril	91270	C_27_H_34_N_2_O_7_	498.6 g/mol	

Angiotensin receptor-blocker					
S. N.	Compound	PubChem ID	Molecular formula	Molecular weight	2D structure
1.	Losartan	11751549	C_22_H_23_ClN_6_O	422.9 g/mol	
2.	Irbesartan	3749	C_25_H_28_N_6_O	428.5 g/mol	
3.	Valsartan	60846	C_24_H_29_N_5_O_3_	435.5 g/mol	
4.	Candesartan	2541	C_24_H_20_N_6_O_3_	440.5 g/mol	
5.	Olmesartan	158781	C_24_H_26_N_6_O_3_	446.5 g/mol	
6.	Telmisartan	65999	C_33_H_30_N_4_O_2_	514.6 g/mol	
7.	Azilsartan	135415867	C_25_H_20_N_4_O_5_	456.4 g/mol	

Diuretics					
S. N.	Compound	PubChem ID	Molecular formula	Molecular weight	2D structure
1.	Hydrochlorothiazide	3639	C_7_H_8_ClN_3_O_4_S_2_	297.7 g/mol	
2.	Chlorthalidone	2732	C_14_H_11_ClN_2_O_4_S	338.8 g/mol	
3.	Metolazone	4170	C_16_H_16_ClN_3_O_3_S	365.8 g/mol	
4.	Furosemide	3440	C_12_H_11_ClN_2_O_5_S	330.74 g/mol	
5.	Bumetanide	2471	C_17_H_20_N_2_O_5_S	364.4 g/mol	

Calcium channel blockers					
S. N.	Compound	PubChem ID	Molecular formula	Molecular weight	2D structure
1.	Amlodipine	2162	C_20_H_25_ClN_2_O_5_	408.9 g/mol	
2.	Clevidipine	153994	C_21_H_23_Cl_2_NO_6_	456.3 g/mol	
3.	Diltiazem	39186	C_22_H_26_N_2_O_4_S	414.5 g/mol	
4.	Felodipine	3333	C_18_H_19_Cl_2_NO_4_	384.2 g/mol	
5.	Nisoldipine	4499	C_20_H_24_N_2_O_6_	388.4 g/mol	
6.	Verapamil	2520	C_27_H_38_N_2_O_4_	454.6 g/mol	

α-Blockers, β-blockers and vasodilators					
S. N.	Compound	PubChem ID	Molecular formula	Molecular weight	2D structure
1.	Nebivolol	71301	C_22_H_25_F_2_NO_4_	405.4 g/mol	
2.	Carvedilol	2585	C_24_H_26_N_2_O_4_	406.5 g/mol	
3.	Nadolol	39147	C_17_H_27_NO_4_	309.4 g/mol	
4.	Propranolol	4946	C_16_H_21_NO_2_	259.339 g/mol	
5.	Bisoprolol	2405	C_18_H_31_NO_4_	325.4 g/mol	
6.	Doxazosin	3157	C_23_H_25_N_5_O_5_	451.5 g/mol	
7.	Prazosin	4893	C_19_H_21_N_5_O_4_	383.4 g/mol	
8.	Terazosin	5401	C_19_H_25_N_5_O_4_	387.4 g/mol	
9.	Minoxidil	4201	C_9_H_15_N_5_O	209.25 g/mol	

Cardiovascular diseases drugs					
S. N.	Compound	PubChem ID	Molecular formula	Molecular weight	2D structure
1.	Atorvastatin	60823	C_33_H_34_FN_2_O_5_^-^	557.6 g/mol	
2.	Fluvastatin	446155	C_24_H_26_FNO_4_	411.5 g/mol	
3.	Pravastatin	4889	C_23_H_36_O_7_	424.5 g/mol	

Anticoagulants and antiplatelet drugs					
S. N.	Compound	PubChem ID	Molecular formula	Molecular weight	2D structure
1.	Aspirin	2244	C_9_H_8_O_4_	180.16 g/mol	
2.	Clopidogrel bisulfate	115366	C_16_H_18_ClNO_6_S_2_	419.9 g/mol	
3.	Warfarin	54678486	C_19_H_16_O_4_	308.3 g/mol	

Anti-arrhythmic drugs					
S. N.	Compound	PubChem ID	Molecular formula	Molecular weight	2D structure
1.	Amiodarone	2157	C_25_H_29_I_2_NO_3_	645.3 g/mol	
2.	Flecainide	3356	C_17_H_20_F_6_N_2_O_3_	414.34 g/mol	
3.	Procainamide	4913	C_13_H_21_N_3_O	235.33 g/mol	
4.	Sotalol	5253	C_12_H_20_N_2_O_3_S	272.37 g/mol	

Cardiac glycosides and drugs used against RTI					
S. N.	Compound	PubChem ID	Molecular formula	Molecular weight	2D structure
1.	Digoxin	2724385	C_41_H_64_O_14_	780.9 g/mol	
2.	Azithromycin	447043	C_38_H_72_N_2_O_12_	749 g/mol	
3.	Amoxicillin	33613	C_16_H_19_N_3_O_5_S	365.4 g/mol	
4.	Doxycycline	54684461	C_22_H_24_N_2_O_8_	444.4 g/mol	
5.	Augmentin	23665637	C_24_H_27_KN_4_O_10_S	602.7 g/mol	
6.	Cephalexin	27447	C_16_H_17_N_3_O_4_S	347.4 g/mol	
7.	Clarithromycin	84029	C_38_H_69_NO_13_	748 g/mol	
8.	Sulfamethoxazole	358641	C_10_H_11_N_3_O_3_S	253.28 g/mol	
9.	Cefuroxime	5479529	C_16_H_16_N_4_O_8_S	424.4 g/mol	
10.	Cefixime	5362065	C_16_H_15_N_5_O_7_S_2_	453.5 g/mol	
11.	Dicloxacillin	18381	C_19_H_17_Cl_2_N_3_O_5_S	470.3 g/mol	

ACE: Angiotensin-converting enzyme; RTI: Respiratory tract infection.

**Table 1C.** Type-III drugs used for *in silico* study against virulent proteins of SARS-CoV-2.

S. N.	Compound	PubChem ID	Molecular formula	Molecular weight	2D structure
1.	Butenafine hydrochloride	443867	C_23_H_28_ClN	353.9 g/mol	
2.	Naftifine	47641	C_21_H_21_N	287.4 g/mol	
3.	Terbinafine	1549008	C_21_H_25_N	291.4 g/mol	
4.	Amphotericin B	5280965	C_47_H_73_NO_17_	924.1 g/mol	
5.	Cinnamaldehyde	637511	C_9_H_8_O	132.16 g/mol	
6.	Citral	638011	C_10_H_16_O	152.23 g/mol	
7.	Caspofungin	2826718	C_52_H_88_N_10_O_15_	1093.3 g/mol	
8.	Micafungin	477468	C_56_H_71_N_9_O_23_S	1270.3 g/mol	
9.	Epigallocatechin gallate	65064	C_22_H_18_O_11_	458.4 g/mol	
10.	Fluconazole	3365	C_13_H_12_F_2_N_6_O	306.27 g/mol	
11.	5-Flucytosine	3366	C_4_H_4_FN_3_O	129.09 g/mol	
12.	Griseofulvin	441140	C_17_H_17_ClO_6_	352.8 g/mol	
13.	Itraconazole	3793	C_35_H_38_Cl_2_N_8_O_4_	705.6 g/mol	
14.	Ketoconazole	456201	C_26_H_28_Cl_2_N_4_O_4_	531.4 g/mol	
15.	Amorolfine	49010	C_21_H_35_NO	317.5 g/mol	
16.	Fenpropimorph	93365	C_20_H_33_NO	303.5 g/mol	
17.	Tridemorph	32518	C_19_H_39_NO	297.5 g/mol	
18.	α-Pinene	6654	C_10_H_16_	136.23 g/mol	
19.	β-Pinene	14896	C_10_H_16_	136.23 g/mol	
20.	1-Aminopiperidine	16658	C_5_H_12_N_2_	100.16 g/mol	
21.	4-Aminopiperidine	424361	C_5_H_12_N_2_	100.16 g/mol	
22.	Piperidine	8082	C_5_H_11_N	85.15 g/mol	
23.	Dithiocarbamate	3037131	CH_2_NS_2_^-^	92.17 g/mol	
24.	Goitrin	3034683	C_5_H_7_NOS	129.18 g/mol	
25.	Tolnaftate	5510	C_19_H_17_NOS	307.4 g/mol	
26.	Rifamycin	6324616	C_37_H_47_NO_12_	697.8 g/mol	
27.	Linezolid	441401	C_16_H_20_FN_3_O_4_	337.35 g/mol	
28.	Neomycin	8378	C_23_H_46_N_6_O_13_	614.6 g/mol	
29.	Tetracycline	54675776	C_22_H_24_N_2_O_8_	444.4 g/mol	
30.	Tigecycline	54686904	C_29_H_39_N_5_O_8_	585.6 g/mol	
31.	Chloramphenicol	5959	C_11_H_12_Cl_2_N_2_O_5_	323.13 g/mol	
32.	Quinupristin	5388937	C_53_H_67_N_9_O_10_S	1022.2 g/mol	
33.	Dalfopristin	6323289	C_34_H_50_N_4_O_9_S	690.8 g/mol	
34.	Geneticin	123865	C_20_H_40_N_4_O_10_	496.6 g/mol	
35.	Clindamycin	446598	C_18_H_33_ClN_2_O_5_S	425 g/mol	
36.	Fusidic acid	3000226	C_31_H_48_O_6_	516.7 g/mol	
37.	Ricin	9821402	C_19_H_36_O_3_	312.5 g/mol	
38.	Puromycin	439530	C_22_H_29_N_7_O_5_	471.5 g/mol	
39.	Virginiamycin	127053480	C_71_H_84_N_10_O_17_	1349.5 g/mol	
40.	Aciclovir	135398513	C_8_H_11_N_5_O_3_	225.2 g/mol	
41.	3-Deaza-adenosine	23190	C_11_H_14_N_4_O_4_	266.25 g/mol	
42.	Arildone	41782	C_20_H_29_ClO_4_	368.9 g/mol	
43.	Hygromycin	6433481	C_23_H_29_NO_12_	511.5 g/mol	
44.	D-glucosamine	439213	C_6_H_13_NO_5_	179.17 g/mol	
45.	Tunicamycin	11104835	C_40_H_66_N_4_O_16_	859 g/mol	
46.	2-Deoxy-D-glucose-[1,2,3H(N)]	91871895	C_6_H_12_O_5_	170.18 g/mol	
47.	Adenosine-5′-[beta, gamma-methylene]triphosphate]	46936826	C_11_H_15_N_5_Na_3_O_13_P_3_	587.15 g/mol	
48.	Foscarnet	3415	CH_3_O_5_P	26.01 g/mol	
49.	Ribavirin	37542	C_8_H_12_N_4_O_5_	244.2 g/mol	
50.	Enviroxime	5361910	C_17_H_18_N_4_O_3_S	358.4 g/mol	
51.	Amantadine	2130	C_10_H_17_N	151.25 g/mol	
52.	Erythromycin	12560	C_37_H_67_NO_13_	733.9 g/mol	
53.	Fidaxomicin	10034073	C_52_H_74_Cl_2_O_18_	1058 g/mol	
54.	Tobramycin	36294	C_18_H_37_N_5_O_9_	467.5 g/mol	
55.	Gentamicin	25200338	C_21_H_43_N_5_O_7_	477.6 g/mol	
56.	Amikacin	37768	C_22_H_43_N_5_O_13_	585.6 g/mol	

The Drug Likeness reports of all the test molecules were prepared by submitting individual SMILES code for each drug to SwissADME – an omic tool aimed at understanding physicochemical description, pharmacokinetics and drug-like properties of small molecules [33,34]. The toxicity and LD_50_ of test compounds were analyzed using the admetSAR 2.0 version.

#### Molecular docking

The binding efficacy of test molecules with the virulent proteins of SARS-CoV-2 was determined by performing docking analysis using iGEMDOCK software (v 2.1), which provides a graphical environment for recognizing pharmacological interactions and virtual screening. The iGEMDOCK generates protein–compound interaction profiles of electrostatic, hydrogen bonding and Van der Waals interactions and infers the pharmacological interactions as well as clusters the screening compounds for the postscreening analysis [35]. On the grounds of evaluated binding energies, it was presumed that how far the drug binds to the target macromolecule. For our study, we used standard docking with a population size of 200, 70 generations and two solutions.

## Results

### Molecular docking analysis

The 3D structures of the SARS-CoV-2 proteins were generated from the SWISS MODEL workspace. Physicochemical and functional characterization of the SARS-CoV-2 structural and nonstructural proteins was analyzed using Expasy’s Prot-param server (Table 2). The structural templates for protein homology modeling were determined using the SWISS MODEL workspace. The template 6zhg.1.C. with 99.68% of sequence identity was observed for homology modeling of spike glycoproteins, while other protein models showed a 100% sequence identity with their respective templates (Table 3).

**Table 2.** Physicochemical and functional characterization of SARS-CoV-2 proteins.

S. N.	Protein	PDB ID	Sequence length	Resolution	MW of protein	pI	Organism
1.	S protein	6VXX	1281	2.8 A°	141410.94	6.09	SARS-CoV-2
2.	E protein	5X29	81	Through NMR	8993.57	8.20	SARS related corona virus
3.	N protein	6M3M	136	2.7 A°	14876.63	9.60	SARS-CoV-2
4.	nsp3	6W6Y	170	1.45 A°	18254.81	6.31	SARS-CoV-2
5.	nsp5	6M03	306	2.0 A°	33796.64	5.95	SARS-CoV-2
6.	nsp10	2G9T	152	2.10 A°	16125.36	5.84	SARS related corona virus
7.	nsp16	6W61	299	1 A°	33454.51	7.61	SARS-CoV-2

CoV: Coronavirus; MW: Molecular weight; NMR: Nuclear magnetic resonance; pI: Isoelectric point.

**Table 3.** Protein validation and homology modeling estimation using the SWISS MODEL workspace.

Protein	Template	Sequence identity (%)	Residues in favorable region (%)	Residues in unfavorable region (%)	C-β deviation	Q-mean score	Mol probity score
S protein	6zgh.1.C	99.68	88.06	2.06	49 (A500-ASN, A410-CYS, A497-THR, A517-GLN, B131-SER, A404-THR, A360-VAL, A131-SER, A525-GLN, B39-THR, A427-ARG, A371-ALA, C497-THR, C234-ASP and A687-THR)	-3.32	1.65
E protein	2 mm4.1.A	100	94.64	0	-	-3.39	1.55
N protein	6y13.1.A	100	96.90	0.78	2 (A11-THR and A65-ASP)	-0.21	1.32
nsp3	6ywk.1.A	100	99.40	0	0	-0.04	0.50
nsp5	6y2g.1.B	100	97.67	0.17	7 (B221-ASN, A113-SER, A154-TYR, B33-ASP, A221-ASN, B282-LEU and B154-TYR)	-0.45	1.05
nsp10	5c8s.1.A	100	97.67	0	-	-2.63	1.68
nsp16	6w61.1.A	100	97.98	0	A98-ASP	-1.34	0.93

The Ramachandran plot depicted structural stability and showed confirmation of residues in the favorable region (Supplementary Figure 1 & Supplementary Table 3). A Ramachandran phi-psi plot for all the seven proteins revealed 88.06–99.40% residues in the allowed region (light grey), and only 0.17–2.06% lay in the disallowed region (white). The above analysis of the predicted structure provides supporting evidence that suggests the predicted 3D structures of SARS-CoV-2 are significant for the docking study. The protein models showed local similarity to the crystal structures of target templates (Supplementary Figure 2). The Q-mean value of protein models was reliable as depicted in the protein homology analysis (Supplementary Figure 3).

**Figure 2.**
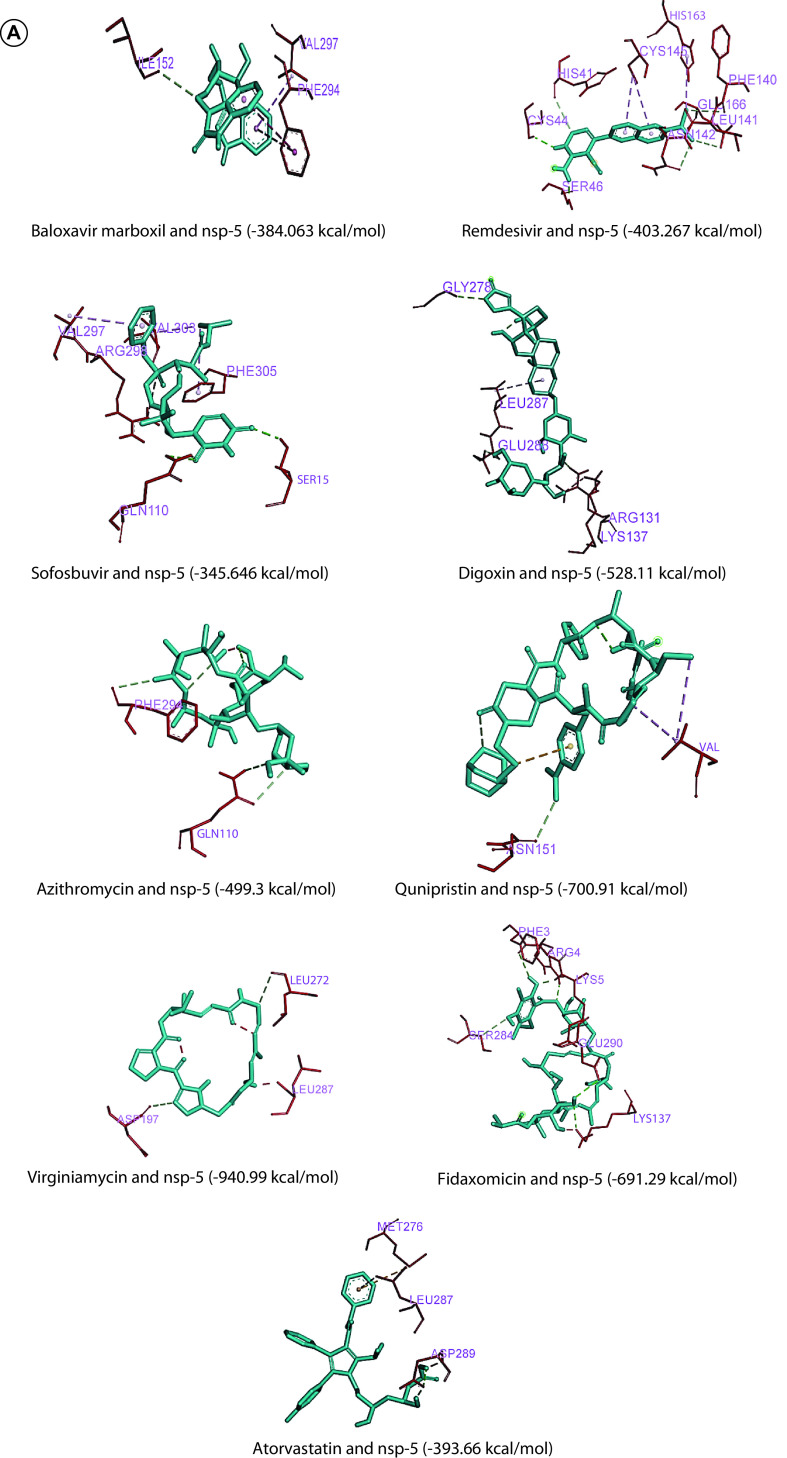

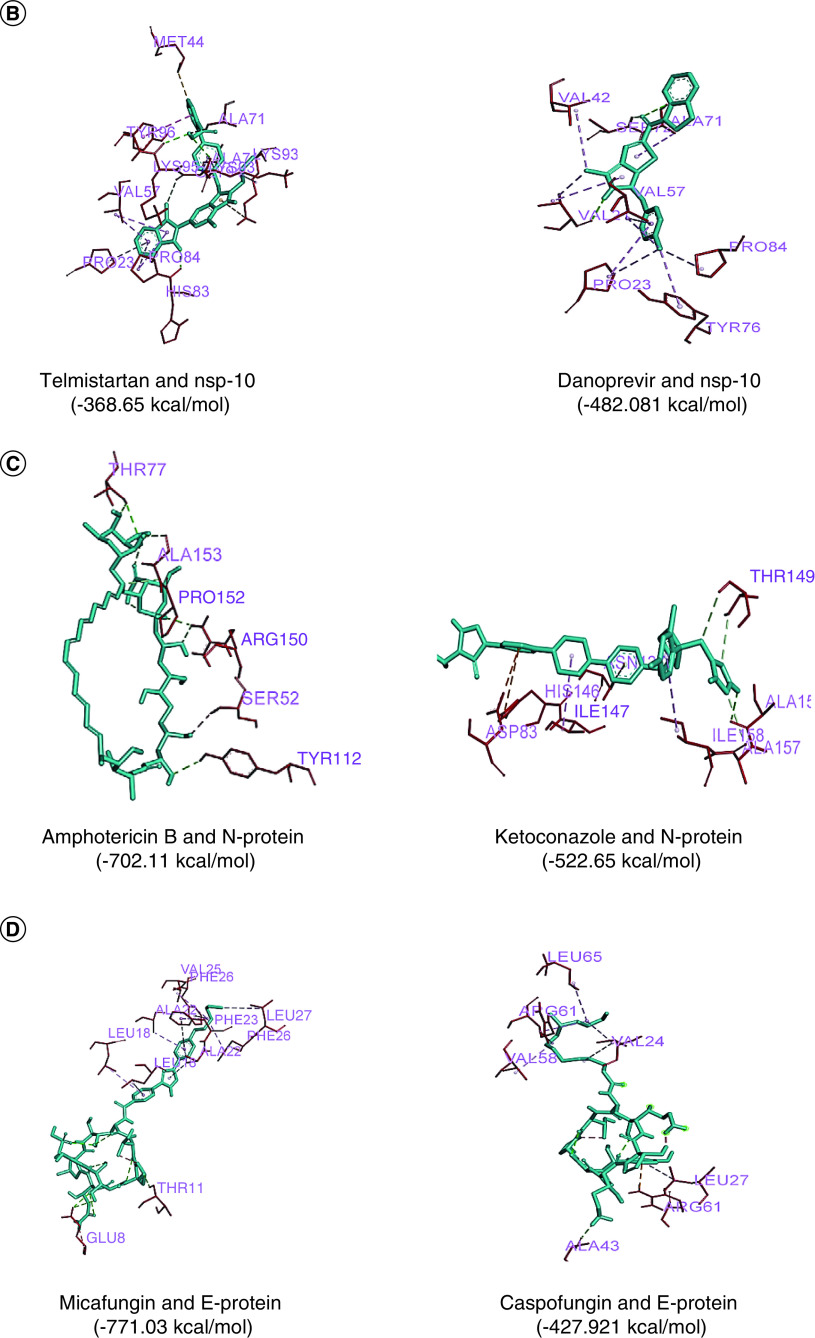
Molecular docking images of interaction between ligands and SARS-CoV-2 proteins. **(A)** Molecular docking images of most efficient interaction between ligands and SARS-CoV-2 nsp5 protein. **(B)** Molecular docking images of most efficient interaction between ligands and SARS-CoV-2 nsp10 protein. **(C)** Molecular docking images of most efficient interaction between ligands and SARS-CoV-2 N-protein. **(D)** Molecular docking images of most efficient interaction between ligands and SARS-CoV-2 E-protein. CoV: Coronavirus.

The molecular docking using iGEMDOCK generated the binding energies of interaction between ligands and the structural PDB proteins of SARS-CoV-2 (Tables 4A, 4B & 4C). The inhibitory potential of all the drugs was assessed based on the binding energy of their interaction with respective proteins (Figure 2A, B, C & D). The favorable docking sites for the interaction between ligands and SARS-CoV-2 proteins were depicted by AADS (Supplementary Table 1).

**Table 4A.** Docking energies (kcal/mol) of interaction between type-I drugs and SARS-CoV-2 target proteins.

S. N.	Proteins Ligands	S-protein	E-protein	N-protein	nsp3	nsp5	nsp10	nsp16
1.	Baloxavir marboxil	-228.062	-364.51	-444.402	-301.679	-384.063	-378.091	-297.187
2.	Baricitinib	-148.24	-236.938	-288.915	-196.09	-249.641	-245.762	-193.172
3.	Chloroquine	-125.43	-200.482	-244.435	-165.922	-211.221	-207.939	-163.456
4.	Cobicistat	-189.883	-226.358	-257.405	-221.819	-255.857	-285.876	-246.005
5.	Danoprevir	-290.779	-436.588	-563.817	-384.631	-489.672	-482.081	-378.918
6.	Darunavir	-216.659	-325.275	-420.101	-286.591	-364.859	-359.199	-282.33
7.	Emtricitabine	-249.332	-277.974	-246.127	-300.418	-351.276	-293.895	-275.723
8.	Favipiravir	-62.717	-93.4829	-122.229	-82.9615	-105.612	-103.973	-81.7278
9.	Hydroxychloroquine	-109.732	-99.3022	-95.1757	-98.7932	-121.876	-98.004	-97.6645
10.	Lopinavir	-200.124	-197.277	-229.527	-282.63	-186.935	-205.436	-164.441
11.	Remdesivir	-239.465	-359.523	-466.708	-316.756	-403.267	-397.008	-312.051
12.	Ribavirin	-96.9263	-154.915	-188.877	-128.218	-163.221	-160.683	-126.307
13.	Ritonavir	-211.267	-220.697	-213.32	-291.495	-210.251	-216.775	-244.912
14.	Sofosbuvir	-205.256	-308.184	-397.956	-271.515	-345.646	-340.27	-267.473
15.	Tenofovir	-114.031	-182.209	-222.242	-150.836	-192.031	-189.05	-148.594
16.	Umifenovir	-140.352	-153.644	-93.3387	-187.772	-108.073	-122.151	-158.747

**Table 4B.** Docking energies (kcal/mol) of interaction between type-II drugs and SARS-CoV-2 target proteins.

S. N.	Proteins Ligands	S-protein	E-protein	N-protein	nsp3	nsp5	nsp10	nsp16
1.	Enalapril	-153.94	-246.04	-2010.23	-203.63	-259.24	-255.21	-200.6
2.	Captolapril	-79.82	-127.58	-135.25	-105.58	-134.42	-132.33	-104.02
3.	Lisinolapril	-126.02	-155.07	-164.01	-176.86	-268.84	-264.67	-137.51
4.	Benezapril	-176.75	-282.49	-264.06	-233.8	-297.64	-293.02	-230.32
5.	Trandolpril	-176.75	-282.5	-248.68	-233.8	-297.64	-293.03	-230.32
6.	Perindopril	-148.24	-236.94	-214.26	-196.1	-249.64	-245.76	-193.17
7.	Fosinopril	-123.55	-250.8	-254.58	-160.52	-384.06	-378.1	-143.15
8.	Ramipril	-171.05	-273.31	-267.34	-226.26	-288.05	-283.56	-222.89
9.	Moexipril	-148.16	-174.09	-267.01	-201.47	-345.65	-340.29	-141.77
10.	Quinapril	-182.45	-291.62	-353.767	-241.35	-307.25	-302.47	-237.75
11.	Losartan	-158.17	-122.45	-175.26	-125.6	-297.65	-293.03	-110.49
12.	Irbesartan	-182.45	-291.61	-265.26	-241.34	-307.26	-302.48	-237.75
13.	Valsartan	-182.45	-291.61	-248.24	-241.34	-307.23	-302.48	-237.75
14.	Candesartan	-154.76	-282.47	-268.13	-128.75	-316.84	-132.36	-142.81
15.	Olmesartan	-146.59	-108.17	-215.03	-145.4	-316.84	-168.24	-108.74
16.	Telmisartan	-139.6	-171.41	-198.94	-261.73	-374.45	-368.65	-167.39
17.	Azilsartan	-193.85	-291.06	-298.28	-256.43	-326.45	-321.38	-252.61
18.	Hydrochlorothiazide	-79.63	-70.81	-84.04	-103.21	-163.22	-88.82	-81.7
19.	Chlorthalidone	-91.75	-91.59	-125	-97.87	-211.23	-101.23	-95.27
20.	Metolazone	-136.84	-218.7	-215	-181	-230.43	-226.85	-178.31
21.	Furosemide	-119.73	-191.36	-168	-158.38	-201.64	-198.49	-156.02
22.	Bumetanide	-84.44	-114.14	-147	-112.85	-240.05	-106.56	-167.75
23.	Amlodipine	-159.64	-255.16	-215.35	-211.18	-268.85	-264.67	-208.03
24.	Clevidipine	-171.05	-256.8	-235.15	-226.26	-288.04	-283.58	-222.89
25.	Diltiazem	-165.34	-264.26	-243.06	-218.71	-278.46	-274.12	-215.46
26.	Felodipine	-142.54	-227.81	-214.27	-188.55	-240.05	-236.31	-185.74
27.	Nisoldipine	-159.64	-255.14	-200.49	-211.18	-268.82	-264.67	-208.03
28.	Verapamil	-140.41	-227.94	-216.34	-213.61	-316.85	-311.94	-116.08
29.	Nebivolol	-133.55	-177.04	-187.14	-135.84	-278.44	-111.75	-140.87
30.	Carvedilol	-108.64	-132.91	-143.25	-148.49	-288.04	-213.22	-163.31
31.	Nadolol	-125.43	-200.49	-172.01	-165.92	-211.23	-107.49	-163.45
32.	Propranolol	-100.7	-114.75	-101.34	-124.13	-182.43	-72.94	-85.97
33.	Bisoprolol	-131.14	-209.58	-231.16	-173.47	-220.83	-217.39	-170.88
34.	Doxazosin	-139.58	-131.05	-214.64	-172.02	-316.85	-221.87	-228.57
35.	Terazosin	-159.64	-255.19	-220.94	-211.18	-268.83	-128.03	-208.03
36.	Hydralazine	-68.42	-109.36	-94.11	-90.5	-115.22	-113.43	-89.16
37.	Minoxidil	-85.52	-136.69	-124.26	-113.13	-144.03	-141.78	-111.45
38.	Atorvastatin	-156.53	-228.89	-215.45	-205.56	-393.66	-220.28	-225.69
39.	Fluvastatin	-156.57	-105.86	-135.15	-155.94	-288.05	-188.96	-120.96
40.	Parvastatin	-171.05	-273.39	-278.11	-226.26	-288.06	-28356	-222.89
41.	Aspirin	-74.12	-111.82	-112.68	-98.05	-124.82	-122.8	-96.59
42.	Clopidogrel bisulfate	-109.53	-157.4	-235.41	-115.93	-249.65	-127.9	-100.4
43.	Warfarin	-131.14	-209.59	-265.36	-173.47	-220.84	-217.4	-170.88
44.	Amiodarone	-142.28	-161.05	-248.11	-156.1	-297.64	-131.8	-225.57
45.	Flecainide	-159.64	-239.7	-215.01	-211.17	-268.85	-264.67	-208.03
46.	Procainamide	-96.93	-154.92	-188.27	-128.21	-163.21	-160.69	-126.3
47.	Sotalol	-102.63	-164.03	-154.15	-135.75	-172.83	-170.14	-133.73
48.	Digoxin	-242.81	-212.72	-265.15	-314.06	-528.11	-320.66	-339.47
49.	Amoxicillin	-142.54	-227.82	-254.39	-188.55	-240.04	-236.29	-185.75
50.	Doxycycline	-188.15	-300.71	-325.87	-248.89	-316.85	-311.94	-245.18
51.	Augmentin	-228.06	-342.41	-254.91	-301.68	-384.05	-377.99	-297.19
52.	Cephalexin	-136.84	-218.71	-264.01	-181.01	-230.44	-226.85	-178.31
53.	Azithromycin	-242.15	-263.78	-258.05	-294.58	-499.3	-308.51	-206.34
54.	Clarithromycin	-240.66	-184.79	-190.06	-261.64	-499.26	-255.59	-212.68
55.	Cefuroxime	-165.34	-264.27	-224.11	-218.72	-278	-274.12	-215.46
56.	Cefixime	-171.05	-273.39	-225.54	-226.26	-288.06	-283.57	-222.89
57.	Sulfamethoxazole	-216.66	-346.29	-301.19	-286.6	-364.86	-359.19	-282.33
58.	Dicloxacillin	-171.05	-273.38	-294.66	-226.26	-288.05	-283.58	-222.89

**Table 4C.** Docking energies (kcal/mol) of interaction between type-III drugs and SARS-CoV-2 target proteins.

S. N.	Proteins Ligands	S-protein	E-protein	N-protein	nsp3	nsp5	nsp10	nsp16
1.	1-Amino piperidine	-40.60	-58.77	-75.37	-53.28	-68.25	-71.43	-52.79
2.	4- Amino piperidine	-45.35	-71.00	-78.06	-56.35	-72.62	-74.51	-53.82
3.	α-Pinene	-38.13	-43.60	-60.47	-54.18	-31.95	-99.42	-27.10
4.	Amorolfine	-103.42	-124.62	-77.7	-108.96	-218.57	-72.27	-99.91
5.	Amphotericine B	-301.25	-391.72	-702.11	-412.79	-245.04	-386	-267.52
6.	β-Pinene	-57.01	-91.13	-111.12	-75.42	-96.01	-99.45	-74.29
7.	Butenafine hydrochloride	-97.92	-106.02	-113.11	-124.31	-119.76	-118.72	-108.14
8.	Caspofungin	-298.74	-427.92	- 327	-292.2	-298	-325	-330.38
9.	Cinnamaldehyde	-56.40	-90.66	-108.91	-75.72	-95.35	-98.72	-72.67
10.	Citral	-45.23	-33.28	-63.39	-47.74	-23.66	-55.48	-51.06
11.	Dithiocarbamate	-25.08	-39.67	-44.28	-31.64	-41.08	-42.16	-30.35
12.	Epigallocatcehin gallate	-83.40	-174.39	-17.25	-161.28	-118.68	-152.2	-191.814
13.	Fenproprimorph	-123.89	-199.54	-240.04	-165.78	-209.04	-216.6	-160.21
14.	Fluconazole	-131.84	-202.21	-239.46	-171.23	-217.09	-221.59	-169.27
15.	5-Flucytosine	-61.33	-92.48	-101.51	-75.28	-93.09	-98.67	-73.99
16.	Goitrin	-41.77	-72.904	-85.38	-61.03	-77.82	-80	-59.19
17.	Griseofulvin	-122.64	-119.80	-56.18	-139.75	-93.51	-244.47	-116.51
18.	Itraconazole	-153.29	-157.55	-523.02	-263.99	-463.65	-96	-175.02
19.	Ketoconazole	-278.61	-412.63	-522.99	-368.66	-463.78	-125	-369.65
20.	Micafungin	-424.67	-771.03	-694	-473.01	-684	-115	-375.32
21.	Naftifine	-124.66	-187.79	-240.88	-164.75	-209.25	-213.6	-161.82
22.	Piperidine	-34.96	-55.19	-64.94	-15.55	-60.2	-61.34	-44.98
23.	Terbinafine	-98.843	-111.47	-66.61	-104.72	-209.26	-88.67	-89.11
24.	Tolnaftate	-123.89	-199.54	-238.58	-165.79	-209.07	-216.21	-160.2
25.	Tridemorph	-118.19	-190.44	-228.94	-158.32	-199.58	-204.27	-152.77
26.	Rifamycin	-171.17	-311.64	-301.14	-273.4	-480.06	-472.63	-310.03
27.	Linezolid	-136.84	-218.7	-185.10	-181	-230.44	-226.85	-178.31
28.	Neomycin	-191.68	-229.63	-264.02	-261.63	-403.26	-220.78	-114.4
29.	Tetracycline	-151.37	-188.99	-116.57	-164.82	-307.25	-198.74	-152.57
30.	Tigecycline	-218.25	-278.52	-198.06	-178.26	-403.22	-351.05	-172.87
31.	Chloramphenicol	-114.03	-182.26	-146.11	-150.84	-192.04	-189.04	-148.59
32.	Quinupristin	-297.34	-311.92	-336.379	-368.05	-700.91	-380.32	-281.08
33.	Dalfopristin	-246.47	-277.18	-265.68	-338.37	-460.89	-312.77	-214.5
34.	Geneticin	-193.85	-309.83	-249.33	-256.43	-326.45	-321.38	-252.61
35.	Clindamycin	-153.94	-246.03	-201.48	-203.64	-259.25	-255.22	-200.6
36.	Fusidic Acid	-192.46	-240.61	-185.24	-214.79	-355.25	-148.98	-168.46
37.	Ricin	-134.93	-135.16	-157.11	-163.59	-268.85	-165.92	-171.91
38.	Puromycin	-151.35	-149.37	-231.92	-143.21	-326.45	-216.18	-156.53
39.	Virginiamycin	-428.28	-399.22	-624.25	-589.65	-940.99	-453.95	-479.18
40.	Aciclovir	-91.22	-69.48	-116.94	-120.67	-153.63	-81	-118.88
41.	3-deaza-adenosine	-56.34	-87.07	-115.45	-97.44	-182.43	-94.41	-77.39
42.	Arildone	-142.54	-227.81	-192.19	-188.54	-240.04	-236.31	-185.75
43.	Hygromycin	-147.28	-201.78	-168.02	-185.82	-345.65	-229.91	-133.34
44.	D-glucosamine	-68.42	-109.35	-92.55	-90.5	-115.22	-113.43	-89.16
45.	Tunicamycin	-222.77	-239.28	-385.75	-265.18	-556.88	-491.97	-254.01
46.	2-Deoxy-D-glucose-[1,2,3H(N)]	-62.72	-100.24	-89.36	-82.96	-105.61	-103.98	-81.73
47.	Adenosine-5′-[beta, gamma-methylene]triphosphate]	-124.9	-157.22	-216.14	-133.41	-297.66	-241.35	-132.92
48.	Foscarnet	-39.91	-59.92	-53.39	-52.79	-67.21	-66.17	-52.01
49.	Ribavirin	-96.93	-154.92	-156.59	-128.22	-163.22	-160.69	-126.31
50.	Enviroxime	-142.54	-227.81	-214.33	-188.55	-240.03	-236.31	-185.75
51.	Amantadine	-62.72	-100.23	-87.15	-82.96	-105.62	-103.97	-81.73
52.	Erythromycin	-235.04	-233.44	-256.28	-293.6	-489.67	-244.59	-232.53
53.	Fidaxomycin	-338.94	-321.37	-450.38	-376.44	-691.29	-646.51	-460.32
54.	Tobramycin	-182.45	-273.94	-265.11	-241.34	-307.25	-302.48	-237.75
55.	Gentamicin	-176.75	-282.5	-268.36	-233.8	-297.65	-293.03	-230.32
56.	Amakicin	-174.13	-178.01	-230.39	-187.86	-384.06	-221.29	-183.59

### ADMET analysis

ADMET parameters, namely ‘absorption’, ‘distribution’, ‘metabolism’ and ‘excretion’, pharmacokinetic properties, and the drug disposition within an organism were determined using Swiss ADME. The LD_50_ dose of all the compounds was estimated by the admetSAR 2.0 version, which is a useful tool for *in silico* screening of ADMET profiles of drug candidates and environmental chemicals.

The ADMET analysis showed moderate to high solubility and gastrointestinal (GI) absorption of antiviral drugs, while baloxavir marboxil, danoprevir and sofosbuvir were found to be soluble but exhibit low GI absorption. Out of 58 drugs studied in the type-II category, fosinopril, telmisartan, amiodarone and azithromycin showed poor solubility and low GI absorption. However, their docking analysis revealed favorable molecular interaction with main protease and nsp10 protein (Supplementary Tables 2A, 2B & 2C). Among the type-III category of drugs, fidaxomycin, micafungin, virginiamycin, tunicamycin, amphotericin B and caspofungin depicted high binding affinity with SARS-CoV-2 main protease, N and E proteins, their ADMET analysis predicted the moderate solubility and low GI absorption.

The lipophilicity of the investigated drugs was indicated between -9.31 and +9.3, except for butenafine hydrochloride (Log p = 0). An *in silico* drug likeliness analysis showed that most of the investigated drugs showed an agreement to the Lipinski, Ghose, Veber, Egan and Muegge rules (Supplementary Tables 2A, 2B & 2C).

The study predicted the bioavailability of drugs based on the ADMET score, which was evaluated between 0.11 and 0.56. The admetSAR 2.0 server was used to predict the rat acute toxicity or LD_50_ value in mol/kg body weight. The study indicated that all the drugs tested against SARS-CoV-2 proteins have LD_50_ values ranged between 0.17 and 4.42 mol/kg (Supplementary Tables 3A, 3B & 3C).

## Discussion

SARS-CoV-2 has emerged as the causal virus for the ongoing COVID-19 pandemic that has spread worldwide. Currently, the cure for COVID-19 is still under trial or observation and multiple researches are ongoing globally to develop a promising vaccine or an antiviral drug. Several drugs like remedesvir and hydroxychloroquine have shown promising results in the treatment of COVID-19 and many more drugs are still under clinical trials. In the current scenario where a completely effective and specific vaccine or drug is still lacking, repurposed drugs could open a new gateway in developing an effective alternative to combat SARS-CoV-2 infection [36].

The SARS-CoV-2 protease is an appealing and important drug target due to its potential involvement in the invasion and replication of the virus. In our study, 15 antiviral drugs with recognized inhibitory potential against different viruses were investigated for their inhibitory activity against SARS-CoV-2. Remdesivir, chloroquine and hydroxychloroquine have been identified as strong inhibitors of the main protease. These drugs are recognized by the WHO among the four potent means of therapeutics against SARS-CoV-2 during the current COVID-19 pandemic [37]. It has been proposed that chloroquine and hydroxychloroquine could alter the endosomal pH and glycosylations of ACE-2 receptors in the host, which blocks binding of SARS-CoV-2 with the altered ACE-2. The *in silico*, *in vitro* and human trials, have proposed the immunomodulatory effect of chloroquine and its derivative hydroxychloroquine in critically ill SARS-CoV-2 infected patients and have indicated their promising role as effective antiviral drugs for the treatment of COVID-19 infections [38,39]. Apart from the aforementioned antiviral drugs, baloxavir marboxil, danoprevir, darunavir and sofosbuvir showed significant interaction with the main protease with the least binding energies, which are -384.06, -489.67, -364.859 and -345.646 kcal/mol, respectively.

Recent *in vitro* studies have demonstrated the role of darunavir in inhibition of viral entry and replication of SARS- CoV-2 via targeting viral protease [26]. However clinical trials for the safety and efficacy of darunavir are still undergoing [40].

The previous *in silico* study has identified RNA-dependent RNA polymerase as the target of sofosbuvir, which is a known antiviral drug used against RNA viruses. In clinical trials, the susceptibility of SARS-CoV-2 to sofosbuvir has been also explored [41]. The clinical efficacy of anti-influenza drugs like baloxavir marboxil and favipiravir was tested *in vitro* against SARS-CoV-2 and has been demonstrated to exhibit inhibitory potential against RNA synthesis [42].

Therefore, based on the previous *in silico, in vitro* and clinical studies as well as the present molecular docking analysis, it can be suggested that besides remdesivir, chloroquine and hydroxychloroquine; other antivirals like baloxavir marboxil, danoprevir and sofosbuvir represent an alternative and a novel drug candidate against SARS-CoV-2. However, drug likeliness report in our investigation also depicted that danoprevir, darunavir, favipiravir and lopinavir are toxic as they have violated three to four rules from Lipinski’s rule of five. The toxicity level of danoprevir could restrict its application as a potent candidate for antiviral therapy; however, the dose regime of the drug is one of the highlighted parameters which is important to be analyzed for the drug toxicity [43].

SARS-CoV-2 enters the host cell by binding to its surface receptors called ACE-2, which are expressed by alveolar epithelial cells [22]. ACE-2 is closely related to ACE, a target of hypertensive drugs. ACE converts angiotensin–I to angiotensin–II, which is known for the narrowing of blood vessels and an increase in blood pressure. Angiotensin-II binds to the target ACE-2 receptors, releasing vasodilatation (angiotensin 1-7). Recent studies have demonstrated the downregulation of ACE-2 in response to viral attachment and deteriorating the health conditions of patients with comorbid conditions like hypertension and RTIs [43,44].

The antihypertensive drugs block angiotensin-I-mediated vasoconstriction and enhance the expression of ACE-2 on the cell surface. This mechanism might impart protection to the lungs and prevents the patients from the high risk associated with SARS-CoV-2 [45,46]. We have investigated the interaction of 58 drugs under the type-II category including ACE-2 inhibitors, ARBs, α- and β-blockers, anticoagulants, and RTI drugs. We identified seven drugs including fosinopril, moexipril, quinapril, telmisartan, azilsartan, verapamil and doxazosin to posses significant interaction with the main protease of SARS-CoV-2. The ACE-2 inhibitors like fosinopril, moexipril and quinapril demonstrated the most significant docking poses with the main protease and N protein with the lowest binding energy.

Furthermore, investigation of docking analysis of ARBs and molecular targets of SARS-CoV-2 showed effective binding affinity of telmisartan, azilsartan, verapamil and doxazosin toward main protease. Recent studies have also depicted the role of antihypertensives such as losartan, olmesartan and telmisartan as antiviral agents against SARS-CoV-2 infection while the clinical trial of telmisartan for COVID-19 therapy has recently started [47,48]. Henceforth, the role of these antihypertensive drugs as promising anti-SARS-CoV-2 drugs cannot be ruled out and could be a subject of future research for assessing their application as repurposed drugs in COVID-19 infection.

The structural and nonstructural viral proteins have the most gruesome role in its infection cycle. Considering their importance in viral attachment, invasion, replication and pathogenicity, we have studied the possible effect of antimicrobial agents targeting the protein or their synthesis. In our computational study, we investigated the binding affinity of 56 such miscellaneous drugs that interacted effectively with the binding site of SARS-CoV-2 proteins. There are previous experimental proofs that have highlighted the application and potential of various antibacterial and antifungal agents targeting intracellular processes like replication, protein synthesis, and cell cycle in viruses like vesicular stomatitis virus, herpes simplex virus types 1, Sindbis virus, influenza virus, vaccinia virus and HIV-1 [49–52].

Antibacterial drugs such as azithromycin, doxycycline, clarithromycin, rifamycin and augmentin (a combination of amoxicillin and clavulanate) are specifically used for the treatment of throat, chest infection and pneumonia. In our study, these antibiotics were identified to exhibit significant binding efficacy at the target site of the main protease (nsp-5) and nsp10. The antiviral potential of several other antibacterial drugs like lymecycline, demeclocycline and eravacycline has been studied *in silico* and *in vitro*, which also indicated the possible application of antibiotics drugs against SARS-CoV-2 [53]. The possible mechanism of the antiviral effect of these antibiotics could lie in the immunopathology of SARS-CoV-2 that resulted in decreased expression of CD-147, which is a transmembrane glycoprotein belonging to the immunoglobulin superfamily. The CD-147 glycoproteins are expressed by epithelial cells, macrophages and type II pneumocytes, and act as an upstream stimulator of matrix metalloproteinases and induce progression of cancer cells. Wang *et al.* have demonstrated CD-147 as an effective receptor SARS-CoV-2 S protein [53]. Antibiotics such as doxycycline and azithromycin have shown reduced expression of CD-147 on carcinoma cell lines, and therefore, these agents could prove to be a possible repurpose drug candidate for SARS-CoV-2 [54].

The present study showed that virginiamycin, tunicamycin, quinipristin, fidaxomycin, digoxin and azithromycin exhibited stable interaction to the residues in the binding pockets of the main protease with the least binding energies. In a previous study, fidaxomicin has also shown effective antiviral efficacy against other viruses like the ZIKA virus and has depicted inhibition of RNA-dependent RNA polymerase and subsequent viral infection *in vitro* and *in vivo* [55]. Antifungal drugs like caspofungin, amphotericin B, ketoconazole and macfungin were found to elicit antiviral effect by showing significant binding affinity toward E and N proteins. In different studies conducted against enterovirus 71, the inhibitory potentials of antifungal drugs like micafungin and amphotericin B were mentioned against enterovirus 71 [50,51].

The spike glycoprotein (S protein) plays an essential role in the wide host tropism of SARS-COV-2 and mediates its pathogenicity via binding to host cell surface receptors and enabling its entry into the host cell [40,41]. Out of 130 drugs, two of the antibiotics namely virginiamycin (-428.28 kcal/mol) and amphotericin B (-301.254 kcal/mol) revealed strong interaction with S protein in the present molecular docking study. Therefore, it is a matter of future investigation to assess whether these antibiotics could prevent cleavage and activation of S1/S2 subunits of S protein and hence could inhibit viral attachment to the host cell receptor.

Mammalian metabolism and toxicity of all the proposed repurposed drugs were tested through ADMET analysis using SWISS-ADME server and admetSAR version 2.0. In terms of drug development; solubility, dissolution and permeability across the GI barrier are the prime focus since drug absorption is important before any associated medical effect can be induced. The solubility and the GI absorption are essential and rate-limiting steps for preformulation interpretations in drug development [56,57]. The potency of any drug is also determined by its ability to reach the blood–brain barrier, which isolates brain tissues from the substances circulating in the blood vascular system. The present study indicated poor membrane permeation properties of all the tested 130 drugs across endothelial capillaries (blood–brain barrier) [58,59].

Permeability glycoprotein (Pgp) or CD243 is an ATP-dependent drug efflux pump extensively distributed and expressed as a receptor on intestinal epithelium that pumps toxins or drugs back into the intestinal lumen and liver [60,61]. CYP450 (CYP3A4) is the main enzyme that catalyzes drug metabolism in the intestine and liver. The co-existence of CYP3A4 and Pgp at the same site synergistically reduces the bioavailability of the drug [62,63]. There was no overlapping observed in CYP3A4 and Pgp suggesting the high bioavailability of the tested drugs.

The study unveils the lipophilic property of all 130 drugs and showed the lipophilic nature of 122 drugs with a positive value of Log P (Log p > 0), while drugs like foscarnet, adenosine-5′-[beta, gamma-methylene] triphosphate, augmentin, minoxidil, hydrochlorothiazide, losartan and fosinopril indicated their hydrophilic nature and higher affinity for the aqueous phase (negative value of Log P). The optimization of pharmacodynamic and pharmacokinetic characteristics of hits and leads in drug development is crucial and is highly dependent on the drug lipophilicity [64,65].

## Conclusion

In the current study, out of 130 drugs that belong to the different class, 15 drugs were identified to be the significant inhibitors of SARS-CoV-2 proteins. The computational study of the target protein and drugs molecular interaction highlighted the potential of baloxavir marboxil, danoprevir, sofosbuvir, fosinopril, quinapril, telmisartan, atrovastatin, sulfamethoxazole, clarithromycin, micafungin, virginiamycin, tunicamycin, fidaxomycin, amphotericin B and caspofungin as a potent inhibitor of SARS-CoV-2 proteins, and therefore, these identified compounds could be explored for their role as future drug candidates against COVID-19.

The biochemical characterization, the pharmacokinetics of the aforementioned potent drugs and the computational analysis of their molecular interactions with SARS-CoV-2 proteins have provided preliminary breakthrough which could lead to exploring their avenues in repurposed drug development. It is pertinent that drug repurposing is an alternative way to curb pandemics like medical emergencies and thus their efficacy to combat SARS-CoV-2 related serious health complications should be explored.

Hence, the effectiveness of baloxavir marboxil, danoprevir, sofosbuvir, fosinopril, quinapril, telmisartan, atrovastatin, sulfamethoxazole, clarithromycin, micafungin, virginiamycin, tunicamycin, fidaxomycin, amphotericin B and caspofungin targeting the structural and nonstructural proteins indicates the possibilities of success of repurposing these drugs against SARS-COV-2. Further *in vitro* and *in vivo* studies of screened compounds alone and their varying combinations could provide an insight into the development and application of these compounds as potent anti-SARS-CoV-2 drugs.

## Future perspective

The study attempted to highlight the inhibitory potential of drugs namely, baloxavir marboxil, danoprevir, sofosbuvir, fosinopril, quinapril, telmisartan, atrovastatin, sulfamethoxazole, clarithromycin, micafungin, virginiamycin, tunicamycin, fidaxomycin, amphotericin B and caspofungin, and thus, revealed their prospects in drug repurposing to combat SARS-CoV-2. The study might lead the way for clinicians, pharmaceutical experts to investigate and further validate these compounds as potent novel and efficacious antiviral therapy against COVID-19 infection.

**Summary Points**

- The study investigated the inhibitory potential and molecular targets of 130 compounds categorized into three types: antivirals (type-I); drugs used in cardiovascular diseases, respiratory tract infection and hypertension (type-II); and antibacterial/antifungal drugs (type-III) against SARS-CoV-2 through computational docking tools.
- Out of 130 compounds, 15 drugs were identified to be significant inhibitors of SARS-CoV-2 and showed a favorable binding affinity with viral S protein, N protein, E- protein, nsp-5, nsp10, nsp-16.
- The identified potent drug candidates such as baloxavir marboxil, danoprevir, sofosbuvir, fosinopril, quinapril, telmisartan, atrovastatin, sulfamethoxazole, clarithromycin, micafungin, virginiamycin, tunicamycin, fidaxomycin, amphotericin B and caspofungin showed promising pharmacokinetic properties and low acute toxicity profile.
- The investigation might lead the way for authenticating the inhibitory potential of these screened compounds by *in vitro* and *in vivo* studies, and further exploring their activity and applications as an individual compound or in varying combinations against SARS-CoV-2.
