# Supplementary material for: Computational guided approach for drug repurposing against SARS-CoV-2
Source: Future Virol. 2021 Mar 2:10.2217/fvl-2020-0403. doi: 10.2217/fvl-2020-0403 (PMC7923689; doi:10.2217/fvl-2020-0403)
Supplement: Supplementary file 1 [file supplementary_files-1.docx]

**
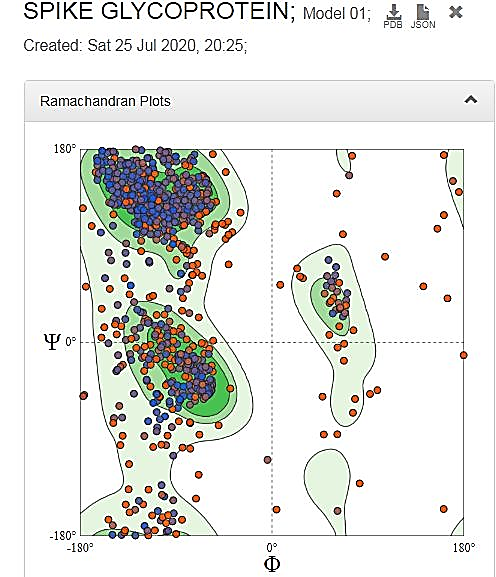

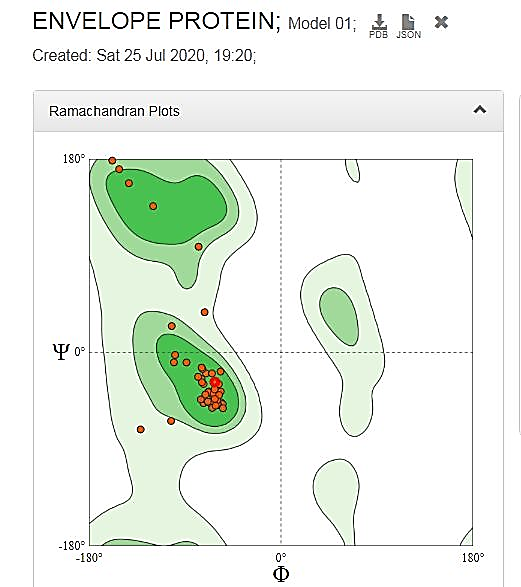

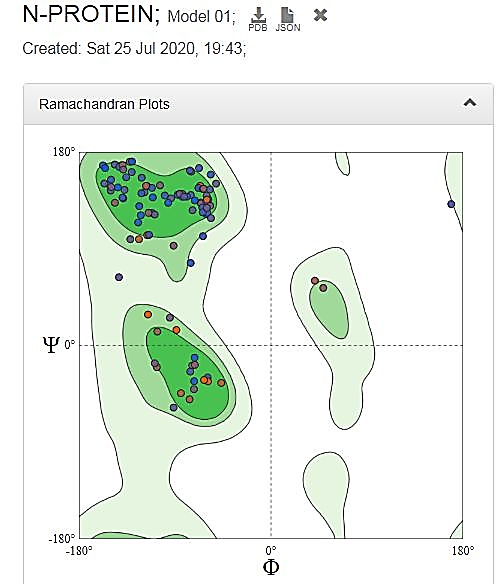
**

**S- protein E- protein N- protein**

**
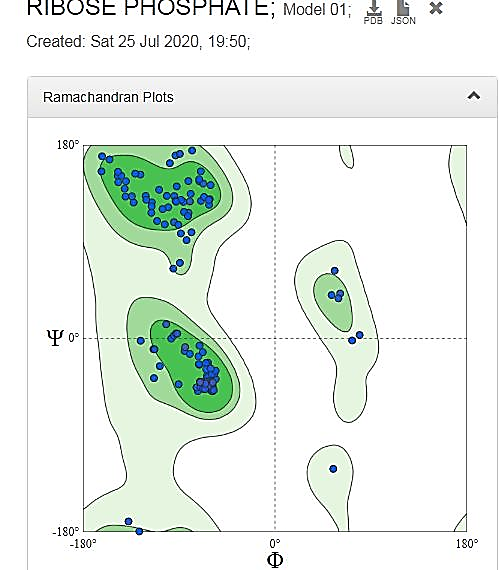

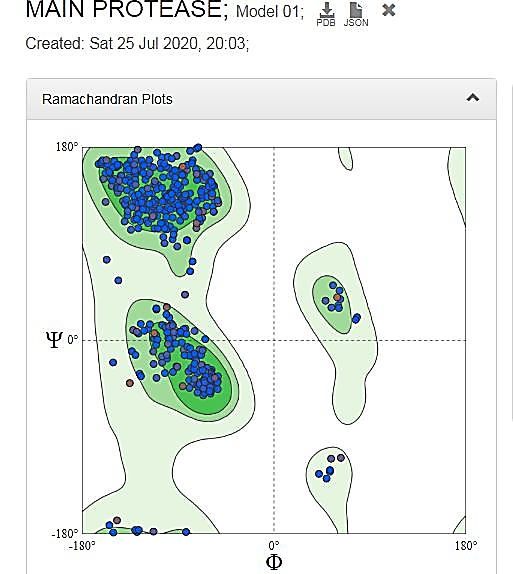
**

**nsp-3 nsp-5**

**
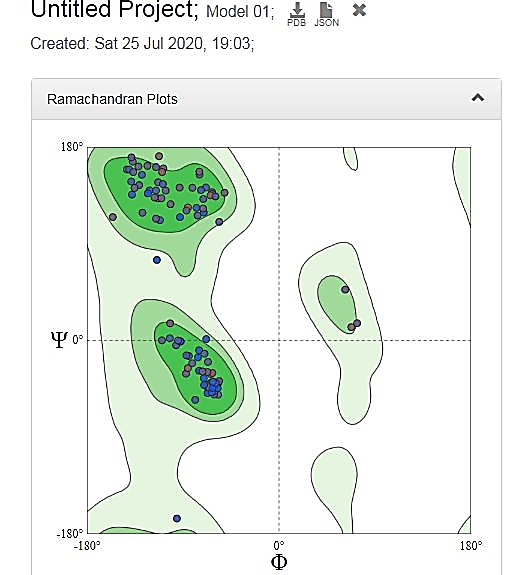

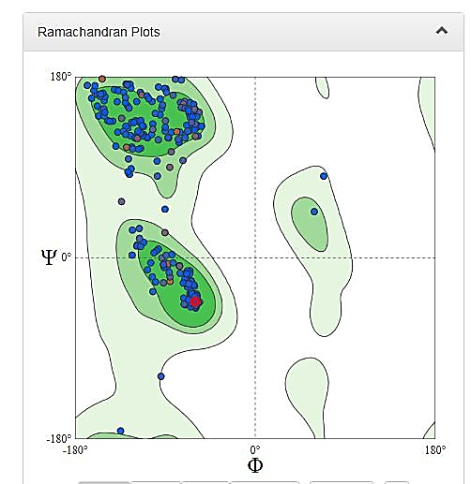
**

**nsp-10 nsp-16**

**Supplementary Figure 1: Ramachandran plot of target SRAS CoV-2 protein homology models.**

**
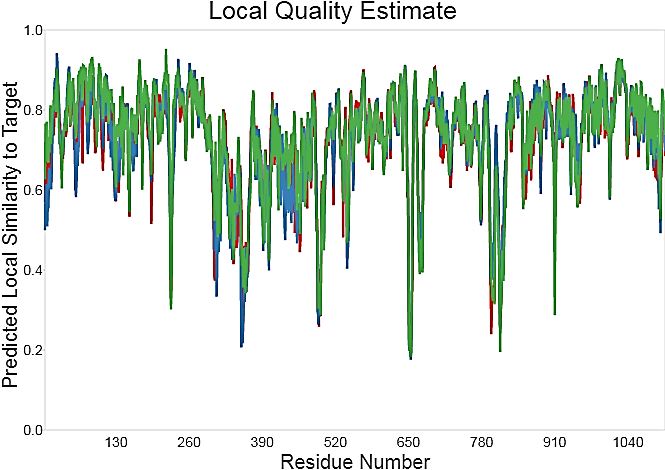

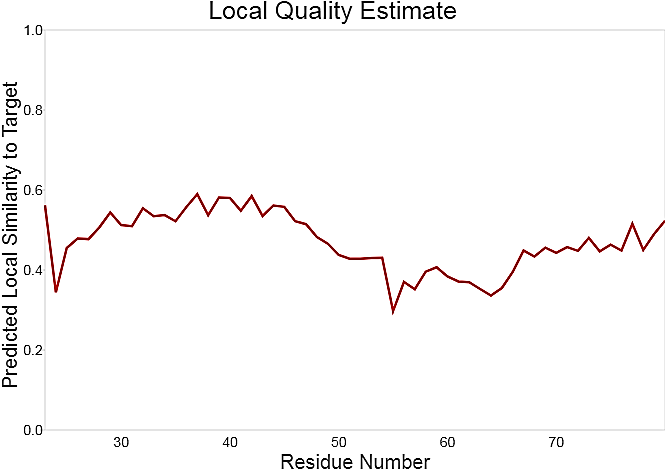
**

**S-protein E-protein**

**
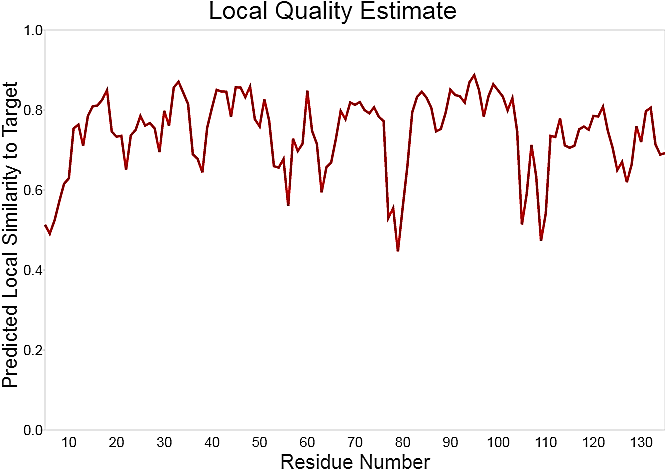

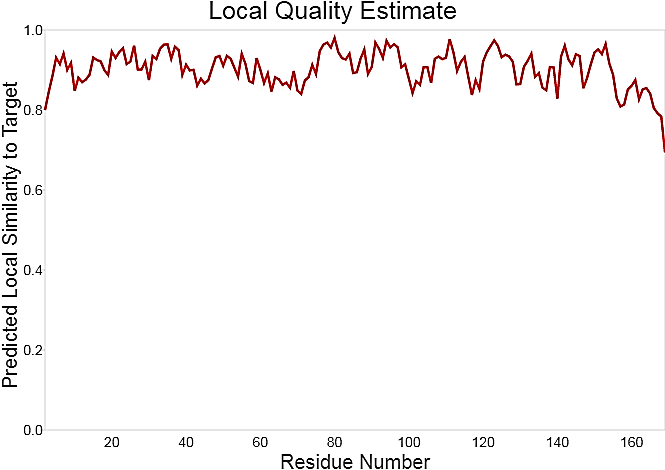
**

**N-protein nsp-3**

**
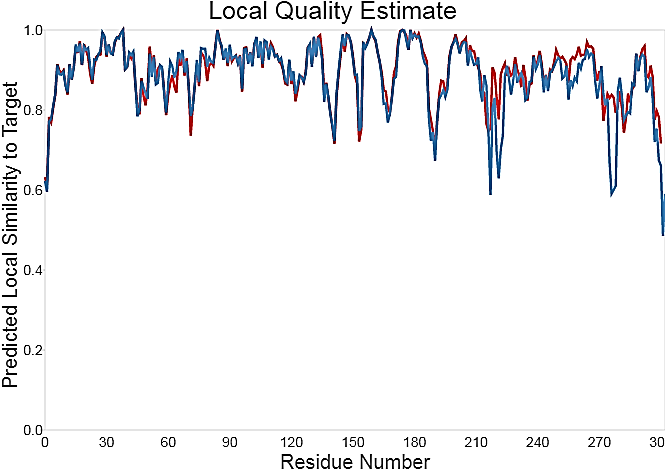

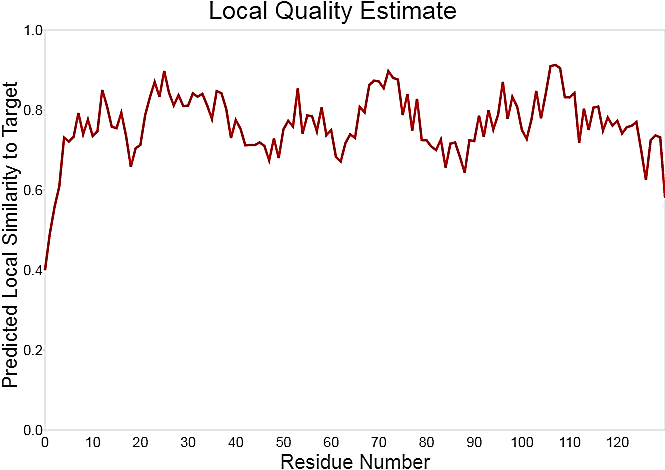
**

**nsp-5 nsp-10**

**
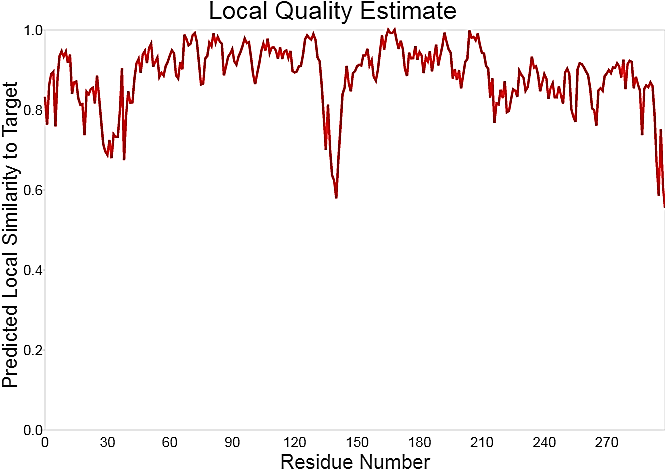
**

**nsp-16**

**Supplementary Figure 2: Local quality estimation of SARS-CoV-2 target proteins.**

**
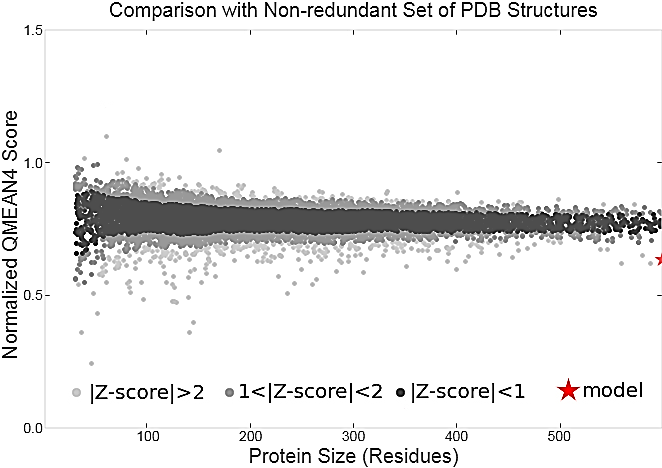

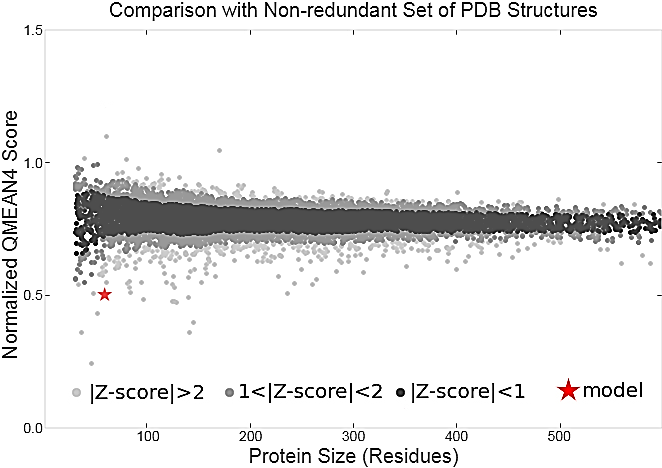
**

**S-protein E- protein**

**
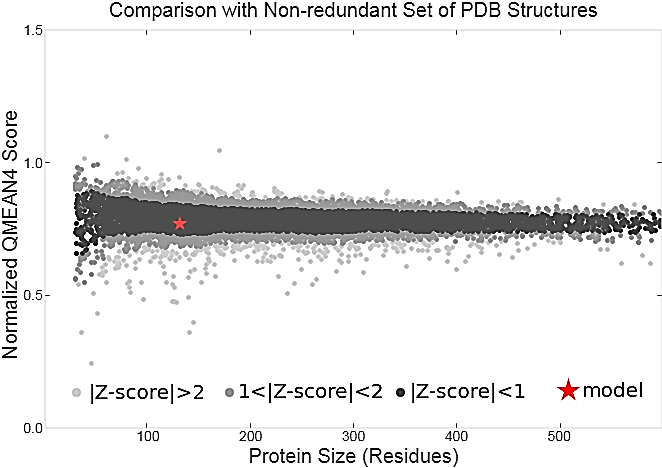

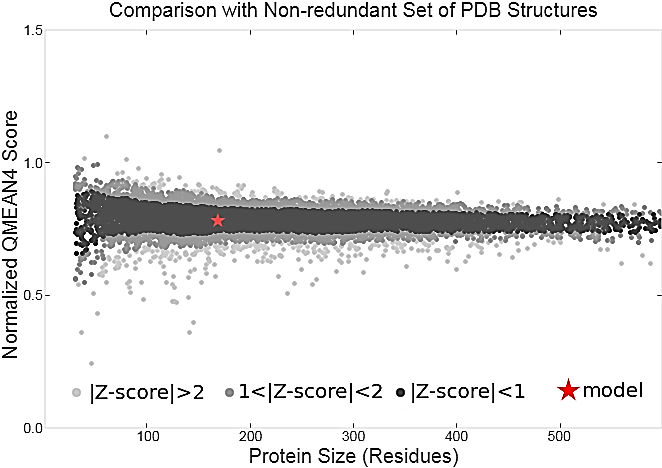
**

**N-protein nsp-3**

**
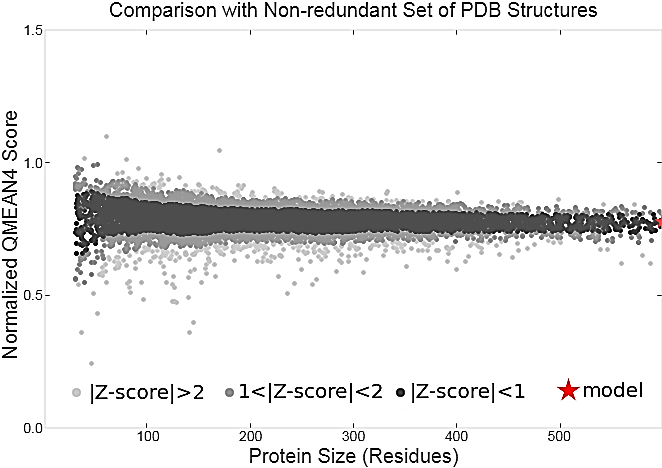

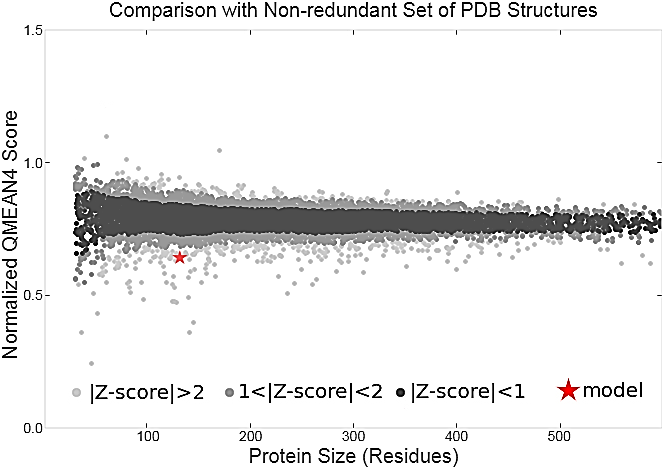
**

**nsp-5 nsp-10**

**
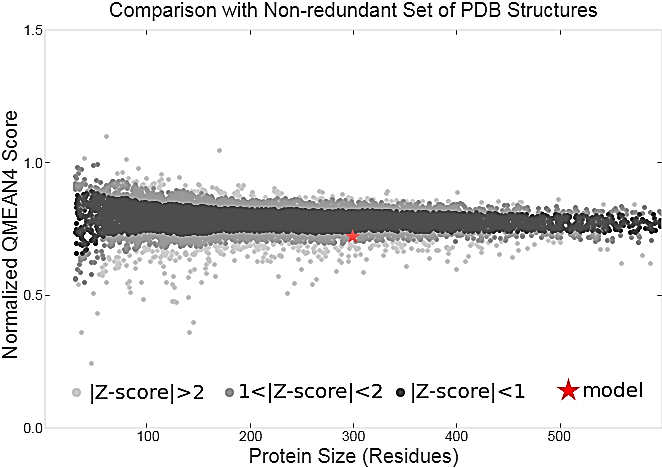
**

**nsp-16**

**Supplementary Figure 3: Q mean Z-score value of SARS-CoV-2 target proteins.**

**Supplementary Table 1: Most Favorable docking sites for the interaction between ligands and SARS-CoV-2 proteins.**

| **S. No.** | **Ligand** | **Protein** | **Favorable amino acid residues at active sites** |
| --- | --- | --- | --- |
|  | **Baloxavir marboxil** | **nsp-5** | **Val- 297, Phe-294, Ile- 152** |
|  | **Remdesivir** |  | **Ser- 49, Cys-44, His-41, His-163, Cys-145, Phe-140, Glu-166, Ile- 141, Asn- 142** |
|  | **Sofosbuvir** |  | **Val- 297, Val- 303, Arg-298, Phe-305, Gln-110, Ser-158** |
|  | **Digoxin** |  | **Gly-278, Glu-288, Arg-131, Lys-137, Leu-287** |
|  | **Azithromycin** |  | **Phe-294, Gln-110** |
|  | **Quinpristin** |  | **Asn-151, Val-303** |
|  | **Virginiamycin** |  | **Asp-197, Leu-272, Leu-287** |
|  | **Fidaxomicin** |  | **Phe-3, Arg-4, Lys-5, Glu-290, Ser-284, Lys-137** |
|  | **Atrovastatin** |  | **Met-276, Leu-287, Asp-289** |
|  | **Danoprevir** | **nsp-10** | **Val-42, Ala-71, Val-42, Val-21, Pro-23, Val-57, Pro-84, Tyr-76** |
|  | **Telmistartan** | **nsp-10** | **Met-44, Tyr- 96, Lys-93, Ala-71, Lys-95, Gly-94, Ala-71, Val-57, Pro-23, His-83, Pro-84** |
|  | **Amphotericin B** | **N-protein** | **Thr-77, Ala-152, Pro-152, Arg-150, Ser-52, Tyr-112** |
|  | **Ketoconoazole** | **N-protein** | **Asp-83, His-146, Ile-147, Asn-127, Ile-158, Ala-157, Ala-156, Thr-145** |
|  | **Caspofungin** | **E-protein** | **Leu-65, Arg-61, Val-24, Val-58, Leu-27, Arg-61, Ala-43** |
|  | **Micafungin** | **E-protein** | **Val-25, The-26, Leu-18, Ala-22, Phe-23, Leu-27, Thr-11, Glu-8, Phe-26, Ala-22** |

**Supplementary Table 2A: Drug-likeliness of type-I compounds (antivirals)**

| **S.No.** | **Molecule** | **Log P** | **ESOL Class** | **GI-ab** | **BBB** | **Lv** | **Gv** | **Vv** | **Ev** | **Mv** | **BS** | **LD50(mol/kg)** |  |
| --- | --- | --- | --- | --- | --- | --- | --- | --- | --- | --- | --- | --- | --- |
|  | Baloxavir marboxil | 3.53 | Moderately soluble | High | No | 1 | 2 | 0 | 0 | 0 | 0.55 | 2.59 |  |
|  | Baricitinib | 1.49 | Very soluble | High | No | 0 | 0 | 0 | 0 | 0 | 0.55 | 2.68 |  |
|  | Chloroquine | 3.95 | Moderately soluble | High | Yes | 0 | 0 | 0 | 0 | 0 | 0.55 | 2.95 |  |
|  | Danoprevir | 4.76 | Moderately soluble | Low | No | 2 | 3 | 2 | 1 | 2 | 0.17 | 2.61 |  |
|  | Darunavir | 9.3 | Insoluble | Low | No | 3 | 4 | 2 | 2 | 7 | 0.17 | 2.60 |  |
|  | Emtricitabine | 5.26 | Very soluble | High | No | 0 | 0 | 0 | 0 | 0 | 0.55 | 2.41 |  |
|  | Favipiravir | 0.7 | Very soluble | High | No | 0 | 3 | 0 | 0 | 1 | 0.55 | 2.15 |  |
|  | Hydroxychloroquine | 2.0 | Soluble | Low | No | 0 | 0 | 0 | 0 | 0 | 0.55 | 2.53 |  |
|  | Lopinavir | 8.81 | Insoluble | Low | No | 3 | 4 | 2 | 2 | 7 | 0.17 | 2.52 |  |
|  | Remdesivir | 3.4 | Moderately soluble | Low | No | 2 | 3 | 2 | 1 | 3 | 0.17 | 2.71 |  |
|  | Ribavirin | 0.13 | Very soluble | Low | No | 0 | 1 | 1 | 1 | 0 | 0.55 | 1.98 |  |
|  | Ritonavir | 4.42 | Poorly soluble | Low | No | 2 | 4 | 2 | 1 | 4 | 0.17 | 2.61 |  |
|  | Sofosbuvir | 3.23 | Soluble | Low | No | 2 | 1 | 2 | 1 | 2 | 0.17 | 2.49 |  |
|  | Tenofovir | 0.24 | Very soluble | Low | No | 0 | 0 | 1 | 1 | 2 | 0.11 | 2.49 |  |
|  | Umifenovir | 3.79 | Moderately soluble | High | No | 0 | 0 | 0 | 0 | 0 | 0.55 | 2.24 | |

**Where, GI-ab is GI absorption, Lv- Lipinski violation, Gv-Ghose violation, Ev- Egan Violation, Mv- Muegge violation, BS- Bioavailability score**

**Supplementary Table 2B. Drug-likeliness of type-II compounds**

| **S.No.** | **Molecule** | **Log P** | **ESOL Class** | **GI-ab** | **BBB** | **Lv** | **Gv** | **Vv** | **Ev** | **Mv** | **BS** | **LD50(mol/kg)** |
| --- | --- | --- | --- | --- | --- | --- | --- | --- | --- | --- | --- | --- |
|  | Enalapril | 3.08 | Very soluble | High | No | 0 | 0 | 1 | 0 | 0 | 0.55 | 1.82 |
|  | Captolpril | 1.46 | Very soluble | High | No | 0 | 0 | 0 | 0 | 0 | 0.56 | 1.74 |
|  | Lisinopril | 2.44 | Highly soluble | High | No | 0 | 0 | 1 | 1 | 1 | 0.55 | 1.87 |
|  | Benezapril | 3.05 | Soluble | High | No | 0 | 0 | 0 | 0 | 0 | 0.55 | 2.20 |
|  | Quinapril | 2.54 | Soluble | High | No | 0 | 0 | 1 | 0 | 0 | 0.55 | 2.26 |
|  | Perindopril | 3.16 | Soluble | High | No | 0 | 0 | 0 | 0 | 0 | 0.55 | 1.94 |
|  | Ramipril | 3.17 | Soluble | High | No | 0 | 0 | 1 | 0 | 0 | 0.55 | 1.67 |
|  | Trandolapril | 3.73 | Soluble | High | No | 0 | 0 | 1 | 0 | 0 | 0.55 | 2.20 |
|  | Fosinopril | -9.31 | Poorly soluble | Low | No | 1 | 3 | 1 | 0 | 2 | 0.55 | 2.86 |
|  | Moexipril | 3.4 | Soluble | High | No | 0 | 2 | 1 | 0 | 0 | 0.55 | 2.51 |
|  | Losartan | -5.4 | Moderately soluble | High | No | 0 | 0 | 0 | 0 | 0 | 0.55 | 2.55 |
|  | Irbesartan | 3.26 | Moderately soluble | High | No | 1 | 1 | 0 | 0 | 0 | 0.55 | 2.77 |
|  | Valsartan | 2.09 | Moderately soluble | High | No | 0 | 0 | 1 | 0 | 0 | 0.56 | 2.65 |
|  | Candesartan | 2.26 | Moderately soluble | High | No | 0 | 0 | 0 | 0 | 0 | 0.56 | 2.55 |
|  | Olmesartan | 2.04 | Moderately soluble | High | No | 0 | 0 | 0 | 0 | 0 | 0.56 | 2.65 |
|  | Telmisartan | 3.88 | Poorly soluble | Low | No | 2 | 3 | 0 | 1 | 1 | 0.56 | 2.80 |
|  | Azilsartan | 2.96 | Moderately soluble | High | No | 0 | 0 | 0 | 0 | 0 | 0.56 | 2.37 |
|  | Hydrochlorothiazide | -0.19 | Very soluble | High | No | 0 | 0 | 0 | 1 | 0 | 0.55 | 2.06 |
|  | Chlorthalidone | 1.52 | Soluble | High | No | 0 | 0 | 0 | 0 | 0 | 0.55 | 1.86 |
|  | Metolazone | 1.91 | Moderately soluble | High | No | 0 | 0 | 0 | 0 | 0 | 0.55 | 1.89 |
|  | Furosemide | 0.85 | Soluble | High | No | 0 | 0 | 0 | 0 | 0 | 0.56 | 2.13 |
|  | Bumetanide | 2.09 | Moderately soluble | High | No | 0 | 0 | 0 | 0 | 0 | 0.56 | 1.81 |
|  | Amlodipine | 3.17 | Soluble | High | No | 0 | 0 | 0 | 0 | 0 | 0.55 | 2.53 |
|  | Clevidipine | 3.55 | Moderately soluble | High | No | 0 | 0 | 0 | 0 | 0 | 0.55 | 2.62 |
|  | Diltiazem | 3.48 | Soluble | High | No | 0 | 0 | 0 | 0 | 0 | 0.55 | 2.41 |
|  | Felodipine | 3.46 | Moderately soluble | High | Yes | 0 | 0 | 0 | 0 | 0 | 0.55 | 2.45 |
|  | Nisoldipine | 3.27 | Soluble | High | No | 0 | 0 | 0 | 0 | 0 | 0.55 | 2.62 |
|  | Verapamil | 4.5 | Moderately soluble | High | Yes | 0 | 2 | 1 | 0 | 0 | 0.55 | 3.41 |
|  | Nebivolol | 3.89 | Moderately soluble | High | Yes | 0 | 0 | 0 | 0 | 0 | 0.55 | 2.72 |
|  | Carvedilol | 3.45 | Moderately soluble | High | Yes | 0 | 0 | 0 | 0 | 0 | 0.55 | 2.42 |
|  | Nadolol | 1.85 | Soluble | High | No | 0 | 0 | 0 | 0 | 0 | 0.55 | 1.79 |
|  | Propranolol | 3.25 | Soluble | High | Yes | 0 | 0 | 0 | 0 | 0 | 0.55 | 2.56 |
|  | Bisoprolol | 4.14 | Soluble | High | Yes | 0 | 0 | 1 | 0 | 0 | 0.55 | 2.00 |
|  | Doxazosin | 3.5 | Moderately soluble | High | No | 0 | 0 | 0 | 0 | 0 | 0.55 | 2.19 |
|  | Prazosin | 2.97 | Soluble | High | No | 0 | 0 | 0 | 0 | 0 | 0.55 | 2.33 |
|  | Terazosin | 2.9 | Soluble | High | No | 0 | 0 | 0 | 0 | 0 | 0.55 | 2.22 |
|  | Minoxidil | -0.51 | Very soluble | High | No | 0 | 0 | 0 | 0 | 0 | 0.55 | 2.43 |
|  | Atorvastatin | 3.81 | Moderately soluble | Low | No | 1 | 4 | 1 | 1 | 0 | 0.56 | 2.56 |
|  | Fluvastatin | 2.89 | Moderately soluble | High | No | 0 | 0 | 0 | 0 | 0 | 0.56 | 2.94 |
|  | Parvastatin | 3.01 | Soluble | High | No | 0 | 0 | 1 | 0 | 0 | 0.56 | 2.60 |
|  | Aspirin | 1.3 | Very soluble | High | Yes | 0 | 0 | 0 | 0 | 1 | 0.56 | 2.62 |
|  | Clopidogrel bisulfate | 2.63 | Soluble | Low | No | 0 | 0 | 1 | 1 | 0 | 0.55 | 2.58 |
|  | Warfarin | 2.41 | Soluble | High | Yes | 0 | 0 | 0 | 0 | 0 | 0.55 | 4.17 |
|  | Amiodarone | 5.26 | Poorly soluble | Low | No | 2 | 3 | 1 | 1 | 2 | 0.17 | 2.65 |
|  | Flecainide | 2.68 | Moderately soluble | High | No | 0 | 0 | 0 | 0 | 0 | 0.55 | 2.56 |
|  | Procainamide | 2.39 | Very soluble | High | Yes | 0 | 0 | 0 | 0 | 0 | 0.55 | 2.11 |
|  | Sotalol | 1.88 | Very soluble | High | No | 0 | 0 | 0 | 0 | 0 | 0.55 | 2.28 |
|  | Digoxin | 4.62 | Moderately soluble | Low | No | 3 | 3 | 1 | 1 | 5 | 0.17 | 4.47 |
|  | Azithromycin | 4.86 | Poorly soluble | Low | No | 2 | 3 | 1 | 1 | 3 | 0.17 | 2.54 |
|  | Amoxicillin | 0.95 | Very soluble | Low | No | 0 | 1 | 1 | 1 | 1 | 0.55 | 1.70 |
|  | Doxycycline | 1.78 | Soluble | Low | No | 2 | 1 | 1 | 1 | 2 | 0.11 | 2.31 |
|  | Augmentin | -8.41 | Very soluble | Low | No | 2 | 3 | 1 | 1 | 4 | 0.17 | 2.16 |
|  | Cephalexin | 1.64 | Soluble | High | No | 0 | 0 | 0 | 1 | 0 | 0.55 | 1.27 |
|  | Clarithromycin | 4.33 | Moderately soluble | Low | No | 2 | 3 | 1 | 1 | 3 | 0.17 | 2.726 |
|  | Bactrim | 2.27 | Moderately soluble | Low | No | 2 | 2 | 1 | 1 | 1 | 0.17 | 2.19 |
|  | Cefuroxime | 2.04 | Very soluble | Low | No | 1 | 1 | 1 | 1 | 1 | 0.11 | 1.65 |
|  | Cefixime | 0.88 | Very soluble | Low | No | 1 | 1 | 1 | 1 | 1 | 0.11 | 1.68 |
|  | Dicloxacillin | 2.42 | Moderately soluble | Low | No | 0 | 0 | 0 | 1 | 0 | 0.56 | 1.99 |

**Where, GI-ab is GI absorption, Lv- Lipinski violation, Gv-Ghose violation, Ev- Egan Violation, Mv- Muegge violation, BS- Bioavailability score**

**Supplementary Table 2C. Drug-likeliness of type-III compounds**

| **S.No.** | **Molecule** | **Log P** | **ESOL Class** | **GI-ab** | **BBB** | **Lv** | **Gv** | **Vv** | **Ev** | **Mv** | **BS** | **LD50(mol/kg)** |
| --- | --- | --- | --- | --- | --- | --- | --- | --- | --- | --- | --- | --- |
|  | 1-aminopiperidine | 1.54 | Very soluble | High | No | 0 | 3 | 0 | 0 | 1 | 0.55 | 2.47 |
|  | 4-aminopiperidine | 1.27 | Very soluble | High | No | 0 | 4 | 0 | 0 | 1 | 0.55 | 2.41 |
|  | Alpha Pinene | 2.63 | Soluble | Low | Yes | 1 | 1 | 0 | 0 | 2 | 0.55 | 1.53 |
|  | Amorolfine | 4.01 | Moderately soluble | High | Yes | 0 | 0 | 0 | 0 | 1 | 0.55 | 2.22 |
|  | Amphotericine B | 3.76 | Moderately soluble | Low | No | 3 | 3 | 1 | 1 | 4 | 0.17 | 2.23 |
|  | Beta Pinene | 2.59 | Soluble | Low | Yes | 1 | 1 | 0 | 0 | 2 | 0.55 | 1.49 |
|  | Butenafine Hydrochloride | 0 | Poorly soluble | Low | No | 1 | 1 | 0 | 1 | 2 | 0.55 | 2.31 |
|  | Caspofungin | 2.97 | Moderately soluble | Low | No | 3 | 4 | 2 | 1 | 5 | 0.17 | 2.93 |
|  | Cinnamaldehyde | 1.65 | Soluble | High | Yes | 0 | 2 | 0 | 0 | 2 | 0.55 | 1.85 |
|  | Citral | 2.47 | Soluble | High | Yes | 0 | 1 | 0 | 0 | 2 | 0.55 | 1.60 |
|  | Dithiocarbamate | 1.0 | Very soluble | High | No | 0 | 3 | 0 | 0 | 2 | 0.56 | 2.70 |
|  | Epigallocatechin gallate | 1.83 | Soluble | Low | No | 2 | 0 | 1 | 1 | 3 | 0.17 | 2.66 |
|  | Fenpropimorph | 4.33 | Moderately soluble | High | Yes | 0 | 0 | 0 | 0 | 0 | 0.55 | 1.99 |
|  | Fluconazole | 0.41 | Soluble | High | No | 0 | 0 | 0 | 0 | 0 | 0.55 | 2.41 |
|  | 5- Flucytosine | 0.39 | Very soluble | High | No | 0 | 3 | 0 | 0 | 2 | 0.55 | 1.94 |
|  | Goitrin | 1.59 | Very soluble | High | No | 0 | 3 | 0 | 0 | 1 | 0.55 | 2.78 |
|  | Griseofulvin | 2.95 | Soluble | High | Yes | 0 | 0 | 0 | 0 | 0 | 0.56 | 1.57 |
|  | Itraconazole | 5.45 | Poorly soluble | High | No | 3 | 3 | 1 | 0 | 2 | 0.17 | 3.31 |
|  | Ketoconazole | 5.45 | Poorly soluble | High | No | 3 | 3 | 1 | 0 | 2 | 0.17 | 3.31 |
|  | Micafungin | 2.65 | Moderately soluble | Low | No | 3 | 4 | 2 | 1 | 5 | 0.11 | 3.47 |
|  | Naftifine | 3.35 | Moderately soluble | High | Yes | 1 | 0 | 0 | 0 | 2 | 0.55 | 2.54 |
|  | Piperidine | 1.70 | Very soluble | Low | No | 0 | 3 | 0 | 0 | 2 | 0.55 | 2.35 |
|  | Terbinafine | 4.15 | Moderately soluble | High | No | 1 | 0 | 0 | 0 | 2 | 0.55 | 1.89 |
|  | Tolnaftate | 3.53 | Moderately soluble | High | Yes | 1 | 0 | 0 | 0 | 1 | 0.55 | 1.74 |
|  | Tridemorph | 5.12 | Moderately soluble | High | Yes | 0 | 0 | 1 | 0 | 1 | 0.55 | 2.62 |
|  | Rifamycin | 4.84 | Poorly soluble | Low | No | 3 | 3 | 1 | 1 | 5 | 0.17 | 2.54 |
|  | Linezolid | 2.31 | Soluble | High | No | 0 | 0 | 0 | 0 | 0 | 0.55 | 2.49 |
|  | Neomycin | 1.26 | Highly soluble | Low | No | 3 | 4 | 1 | 1 | 5 | 0.17 | 1.48 |
|  | Tetracycline | 1.41 | Very soluble | Low | No | 1 | 0 | 1 | 1 | 2 | 0.11 | 2.69 |
|  | Tigecycline | 2.26 | Soluble | Low | No | 3 | 3 | 1 | 1 | 2 | 0.11 | 2.68 |
|  | Chloramphenicol | 1.17 | Soluble | High | No | 0 | 0 | 0 | 0 | 0 | 0.55 | 2.22 |
|  | Quinupristin | 4.06 | Poorly soluble | Low | No | 2 | 3 | 2 | 1 | 4 | 0.17 | 2.93 |
|  | Dalfopristin | 3.26 | Moderately soluble | Low | No | 2 | 3 | 1 | 1 | 3 | 0.17 | 2.60 |
|  | Geneticin | 2.08 | Highly soluble | Low | No | 2 | 3 | 1 | 1 | 4 | 0.17 | 1.90 |
|  | Clindamycin | 3.16 | Soluble | High | No | 0 | 0 | 0 | 0 | 0 | 0.55 | 2.31 |
|  | Fusidic Acid | 3.6 | Poorly soluble | Low | No | 1 | 4 | 0 | 0 | 1 | 0.56 | 3.59 |
|  | Ricin | 3.1 | Soluble | High | No | 0 | 0 | 0 | 0 | 0 | 0.55 | 2.89 |
|  | Puromycin | 3.11 | Soluble | Low | No | 1 | 1 | 1 | 1 | 1 | 0.55 | 2.26 |
|  | Virginiamycin | 0.88 | Insoluble | Low | No | 3 | 3 | 1 | 1 | 6 | 0.17 | 2.90 |
|  | Acyclovir | 0.48 | Very soluble | High | No | 0 | 1 | 0 | 0 | 0 | 0.55 | 1.52 |
|  | 3-deaza-adenosine | 0.62 | Very soluble | Low | No | 0 | 1 | 0 | 0 | 0 | 0.55 | 1.97 |
|  | Arildone | 4.25 | Moderately soluble | High | Yes | 0 | 0 | 1 | 0 | 1 | 0.55 | 2.51 |
|  | Hygromycin | 0.75 | Very soluble | Low | No | 3 | 2 | 1 | 1 | 3 | 0.17 | 2.45 |
|  | D-glucosamine | 0.21 | Highly soluble | Low | No | 0 | 2 | 0 | 0 | 2 | 0.55 | 1.22 |
|  | Tunicamycin | 3.25 | Soluble | Low | No | 3 | 4 | 2 | 1 | 5 | 0.17 | 2.51 |
|  | 2-Deoxy-D-glucose-[1,2,3H(N)] | 0.65 | Highly soluble | High | No | 0 | 2 | 0 | 0 | 1 | 0.55 | 1.04 |
|  | Adenosine-5'-[beta, gamma-methylene]triphosphate | -1.43 | Highly soluble | Low | No | 3 | 2 | 1 | 1 | 4 | 0.11 | 2.59 |
|  | Foscarnet | -1.02 | Highly soluble | High | No | 0 | 3 | 0 | 0 | 2 | 0.56 | 1.71 |
|  | Ribavirin | 0.35 | Very soluble | Low | No | 0 | 1 | 1 | 1 | 0 | 0.55 | 1.98 |
|  | Enviroxime | 2.37 | Moderately soluble | High | No | 0 | 0 | 0 | 0 | 0 | 0.55 | 2.37 |
|  | Amantadine | 1.91 | Soluble | High | Yes | 0 | 1 | 0 | 0 | 2 | 0.55 | 2.25 |
|  | Erythromycin | 5.11 | Moderately soluble | Low | No | 2 | 3 | 1 | 1 | 3 | 0.17 | 2.22 |
|  | Fidaxomycin | 6.23 | Poorly soluble | Low | No | 3 | 4 | 2 | 2 | 5 | 0.17 | 2.67 |
|  | Tobramycin | 1.46 | Highly soluble | Low | No | 2 | 1 | 1 | 1 | 4 | 0.17 | 1.76 |
|  | Gentamicin | 2.08 | Highly soluble | Low | No | 2 | 2 | 1 | 1 | 3 | 0.17 | 1.89 |
|  | Amakicin | 1.19 | Highly soluble | Low | No | 3 | 3 | 2 | 1 | 4 | 0.17 | 1.75 |

**Where, GI-ab is GI absorption, Lv- Lipinski violation, Gv-Ghose violation, Ev- Egan Violation, Mv- Muegge violation, BS- Bioavailability score**

**Supplementary Table 3A: Interaction of type-I compounds with cytochromes P450 (CYP) and permeability glycoproteins.**

| **S.No.** | **Molecule** | **Pgp substrate** | **CYP1A2 inhibitor** | **CYP2C19 inhibitor** | **CYP2C9 inhibitor** | **CYP2D6 inhibitor** |
| --- | --- | --- | --- | --- | --- | --- |
|  | Baloxavir marboxil | Yes | No | No | Yes | Yes |
|  | Baricitinib | Yes | No | No | No | No |
|  | Chloroquine | No | Yes | No | No | Yes |
|  | Danoprevir | Yes | No | No | No | No |
|  | Darunavir | Yes | No | No | No | No |
|  | Emtricitabine | No | No | No | No | No |
|  | Favipiravir | No | No | No | No | No |
|  | Hydroxychloroquine | Yes | No | No | No | No |
|  | Lopinavir | Yes | No | No | No | No |
|  | Remdesivir | Yes | No | No | No | No |
|  | Ribavirin | No | No | No | No | No |
|  | Ritonavir | Yes | No | No | No | No |
|  | Sofosbuvir | Yes | No | No | No | No |
|  | Tenofovir | No | No | No | No | No |
|  | Umifenovir) | No | No | Yes | Yes | Yes |

**Supplementary Table 3B: Interaction of type-II compounds with cytochromes P450 (CYP) and permeability glycoproteins.**

|  | **ARB** | | | | | |
| --- | --- | --- | --- | --- | --- | --- |
| **S.No.** | **Molecule** | **Pgp substrate** | **CYP1A2 inhibitor** | **CYP2C19 inhibitor** | **CYP2C9 inhibitor** | **CYP2D6 inhibitor** |
|  | Losartan | Yes | No | Yes | No | No |
|  | Irbesartan | Yes | Yes | Yes | Yes | Yes |
|  | Valsartan | No | No | Yes | Yes | No |
|  | Candesartan | Yes | Yes | Yes | Yes | No |
|  | Olmesartan | Yes | No | Yes | No | No |
|  | Telmisartan | No | No | Yes | No | No |
|  | Azilsartan | No | No | Yes | Yes | No |

|  | **ACE INHIBITTORS** | | | | | |
| --- | --- | --- | --- | --- | --- | --- |
| **S.No.** | **Molecule** | **Pgp substrate** | **CYP1A2 inhibitor** | **CYP2C19 inhibitor** | **CYP2C9 inhibitor** | **CYP2D6 inhibitor** |
|  | Enalapril | Yes | No | No | No | No |
|  | Captolpril | No | No | No | No | No |
|  | Lisinopril | Yes | No | No | No | No |
|  | Benezapril | Yes | No | No | No | Yes |
|  | Quinapril | Yes | No | No | No | No |
|  | Perindopril | Yes | No | No | No | No |
|  | Ramipril | Yes | No | No | No | Yes |
|  | Trandolapril | Yes | No | No | No | Yes |
|  | Fosinopril | Yes | No | No | No | No |
|  | Moexipril | Yes | No | No | No | Yes |

|  | **DIURETICS** | | | | | |
| --- | --- | --- | --- | --- | --- | --- |
| **S.No.** | **Molecule** | **Pgp substrate** | **CYP1A2 inhibitor** | **CYP2C19 inhibitor** | **CYP2C9 inhibitor** | **CYP2D6 inhibitor** |
|  | Hydrochlorothiazide | No | No | No | No | No |
|  | Chlorthalidone | No | No | No | No | No |
|  | Metolazone | Yes | No | No | No | No |
|  | Furosemide | No | No | No | No | No |
|  | Bumetanide | No | No | No | Yes | No |

|  | **CCB** | | | | | |
| --- | --- | --- | --- | --- | --- | --- |
| **S.No.** | **Molecule** | **Pgp substrate** | **CYP1A2 inhibitor** | **CYP2C19 inhibitor** | **CYP2C9 inhibitor** | **CYP2D6 inhibitor** |
|  | Amlodipine | Yes | Yes | Yes | Yes | No |
|  | Clevidipine | No | Yes | Yes | Yes | Yes |
|  | Diltiazem | No | No | Yes | Yes | Yes |
|  | Felodipine | No | Yes | Yes | Yes | No |
|  | Nisoldipine | No | Yes | Yes | Yes | Yes |
|  | Verapamil | Yes | No | No | No | Yes |

|  | **ALPHA BLOCKERS, BETA BLOCKERS and VASODILATORS** | | | | | |
| --- | --- | --- | --- | --- | --- | --- |
| **S.No.** | **Molecule** | **Pgp substrate** | **CYP1A2 inhibitor** | **CYP2C19 inhibitor** | **CYP2C9 inhibitor** | **CYP2D6 inhibitor** |
|  | Nebivolol | Yes | No | No | No | Yes |
|  | Carvedilol | Yes | Yes | Yes | Yes | Yes |
|  | Nadolol | Yes | No | No | No | No |
|  | Propranolol | No | Yes | No | No | Yes |
|  | Bisoprolol | Yes | No | No | No | Yes |
|  | Doxazosin | No | No | No | Yes | Yes |
|  | Prazosin | Yes | No | No | Yes | Yes |
|  | Terazosin | Yes | Yes | No | No | Yes |
|  | Minoxidil | No | No | No | No | No |

|  | **CARDIOVASCULAR DRUGS** | | | | | |
| --- | --- | --- | --- | --- | --- | --- |
| **S.No.** | **Molecule** | **Pgp substrate** | **CYP1A2 inhibitor** | **CYP2C19 inhibitor** | **CYP2C9 inhibitor** | **CYP2D6 inhibitor** |
|  | Atorvastatin | Yes | No | Yes | No | Yes |
|  | Fluvastatin | Yes | No | No | Yes | No |
|  | Parvastatin | Yes | No | No | No | No |

|  | **ANTIARRYTHMIC DRUGS** | | | | | |
| --- | --- | --- | --- | --- | --- | --- |
| **S.No.** | **Molecule** | **Pgp substrate** | **CYP1A2 inhibitor** | **CYP2C19 inhibitor** | **CYP2C9 inhibitor** | **CYP2D6 inhibitor** |
|  | Amiodarone | Yes | No | No | No | No |
|  | Flecainide | No | No | No | No | Yes |
|  | Procainamide | No | No | No | No | No |
|  | Sotalol | No | No | No | No | No |

|  | **ANTICOAGULANTS AND ANTIPLATELET** | | | | | |
| --- | --- | --- | --- | --- | --- | --- |
| **S.No.** | **Molecule** | **Pgp substrate** | **CYP1A2 inhibitor** | **CYP2C19 inhibitor** | **CYP2C9 inhibitor** | **CYP2D6 inhibitor** |
|  | Aspirin | No | No | No | No | No |
|  | Clopidogrel bisulfate | Yes | No | No | No | No |
|  | Warfarin | No | No | Yes | Yes | No |

|  | **CARDIAC GLYCOSIDES AND DRUGS AGAINST RTI** | | | | | |
| --- | --- | --- | --- | --- | --- | --- |
| **S.No.** | **Molecule** | **Pgp substrate** | **CYP1A2 inhibitor** | **CYP2C19 inhibitor** | **CYP2C9 inhibitor** | **CYP2D6 inhibitor** |
|  | Digoxin | Yes | No | No | No | No |
|  | Azithromycin | Yes | No | No | No | No |
|  | Amoxicillin | No | No | No | No | No |
|  | Doxycycline | Yes | No | No | No | No |
|  | Augmentin | No | No | No | No | No |
|  | Cephalexin | Yes | No | No | No | No |
|  | Azithromycin | Yes | No | No | No | No |
|  | Clarithromycin | Yes | No | No | No | No |
|  | Bactrim | No | No | No | No | No |
|  | Cefuroxime | No | No | No | No | No |
|  | Cefixime | No | No | No | No | No |
|  | Dicloxacillin | Yes | No | Yes | No | No |

**Supplementary Table 3C: Interaction of type-III compounds with cytochromes P450 (CYP) and permeability glycoproteins.**

| **S.No.** | **Molecule** | **Pgp substrate** | **CYP1A2 inhibitor** | **CYP2C19 inhibitor** | **CYP2C9 inhibitor** | **CYP2D6 inhibitor** |
| --- | --- | --- | --- | --- | --- | --- |
|  | 1-aminopiperidine | No | No | No | No | No |
|  | 4-aminopiperidine | No | No | No | No | No |
|  | Alpha Pinene | No | No | No | No | No |
|  | Amorolfine | No | No | No | Yes | No |
|  | Amphotericine B | Yes | No | No | No | No |
|  | Beta Pinene | No | No | No | No | No |
|  | Butenafine Hydrochloride | Yes | No | No | Yes | No |
|  | Caspofungin | No | No | No | No | No |
|  | Cinnamaldehyde | No | No | No | No | No |
|  | Citral | No | No | No | No | No |
|  | Dithiocarbamate | No | No | No | No | No |
|  | EGCG | No | No | No | No | No |
|  | Fenpropimorph | No | No | No | Yes | No |
|  | Fluconazole | No | No | Yes | No | No |
|  | Flucytosine | No | No | No | No | No |
|  | Goitrin | No | No | No | No | No |
|  | Griseofulvin | No | Yes | No | No | Yes |
|  | Itraconazole | Yes | Yes | Yes | Yes | Yes |
|  | Ketoconazole | Yes | Yes | Yes | Yes | Yes |
|  | Micafungin | Yes | No | No | No | No |
|  | Naftifine | No | Yes | Yes | Yes | No |
|  | Piperidine | No | No | No | No | No |
|  | Terbinafine | No | No | No | Yes | No |
|  | Tolnaftate | No | Yes | Yes | No | No |
|  | Tridemorph | No | Yes | No | Yes | No |
|  | Rifamycin | Yes | No | No | No | No |
|  | Linezolid | Yes | No | No | No | No |
|  | Neomycin | Yes | No | No | No | No |
|  | Tetracycline | No | No | No | No | No |
|  | Tigecycline | Yes | No | No | No | No |
|  | Chloramphenicol | No | No | No | No | No |
|  | Quinupristin | Yes | No | No | No | No |
|  | Dalfopristin | Yes | No | No | No | No |
|  | Geneticin | Yes | No | No | No | No |
|  | Clindamycin | Yes | No | No | No | No |
|  | Fusidic Acid | Yes | No | No | No | No |
|  | Ricin | Yes | No | No | No | No |
|  | Puromycin | Yes | No | No | No | No |
|  | Virginiamycin | Yes | No | No | No | No |
|  | Aciclovir | No | No | No | No | No |
|  | 3-deaza-adenosine | No | No | No | No | No |
|  | Arildone | No | Yes | No | No | Yes |
|  | Hygromycin | No | No | No | No | No |
|  | D-glucosamine | Yes | No | No | No | No |
|  | Tunicamycin | Yes | No | No | No | No |
|  | 2-Deoxy-D-glucose-[1,2,3H(N)] | No | No | No | No | No |
|  | Adenosine-5'-[beta, gamma-methylene]triphosphate | No | No | No | No | No |
|  | Foscarnate | No | No | No | No | No |
|  | Ribavirin | No | No | No | No | No |
|  | Enviroxime | No | No | No | Yes | No |
|  | Amantadine | No | No | No | No | No |
|  | Erythromycin | Yes | No | No | No | No |
|  | Fidaxomycin | Yes | No | No | No | No |
|  | Tobramycin | Yes | No | No | No | No |
|  | Gentamicin | Yes | No | No | No | No |
|  | Amakicin | Yes | No | No | No | No |
